# Luminescence Lifetime-Based Sensing Platform Based on Cyclometalated Iridium(III) Complexes for the Detection of Perfluorooctanoic Acid in Aqueous Samples

**DOI:** 10.1021/acs.analchem.3c04289

**Published:** 2024-01-16

**Authors:** Kun Zhang, Andrew J. Carrod, Elena Del Giorgio, Joseph Hughes, Knut Rurack, Francesca Bennet, Vasile-Dan Hodoroaba, Stuart Harrad, Zoe Pikramenou

**Affiliations:** † School of Chemistry, University of Birmingham, Birmingham B15 2TT, U.K.; ‡ School of Geography, Earth & Environmental Sciences, University of Birmingham, Birmingham B15 2TT, U.K.; § Chemical and Optical Sensing Division, Federal Institute for Materials Research and Testing (BAM), Richard-Willstätter-Str. 11, 12489 Berlin, Germany; ∥ Surface Analysis and Interfacial Chemistry Division, Federal Institute for Materials Research and Testing (BAM), Unter den Eichen 44-46, 12203 Berlin, Germany

## Abstract

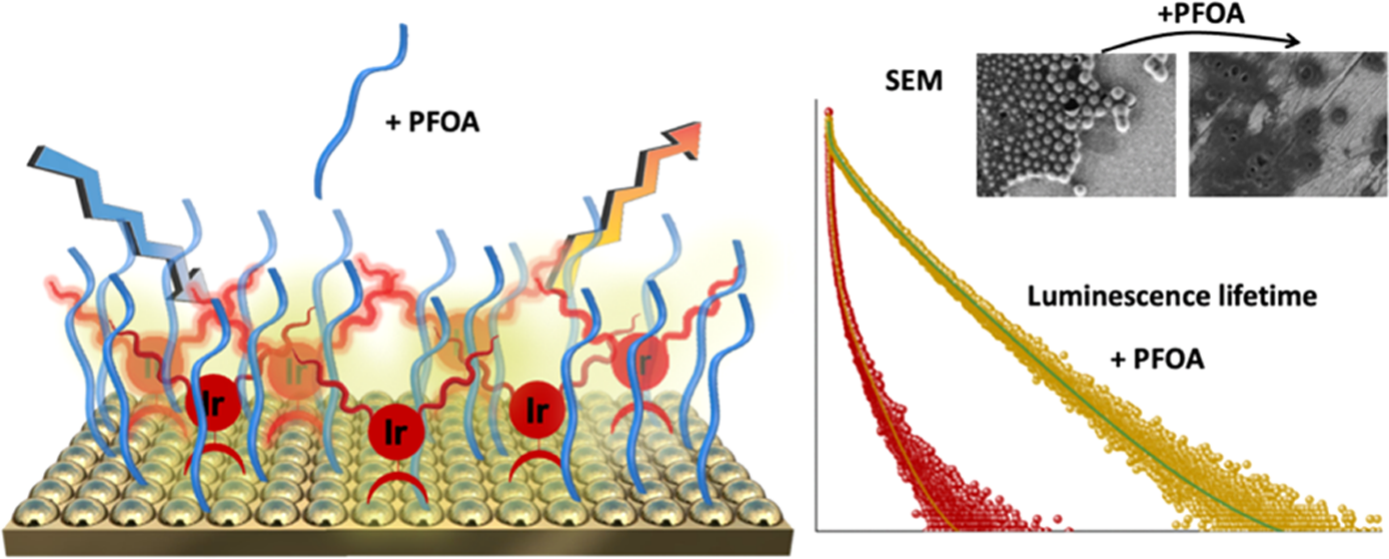

Luminescence lifetimes are an attractive analytical method for detection due to its high sensitivity and stability. Iridium probes exhibit luminescence with long excited-state lifetimes, which are sensitive to the local environment. Perfluorooctanoic acid (PFOA) is listed as a chemical of high concern regarding its toxicity and is classified as a “forever chemical”. In addition to strict limits on the presence of PFOA in drinking water, environmental contamination from industrial effluent or chemical spills requires rapid, simple, accurate, and cost-effective analysis in order to aid containment. Herein, we report the fabrication and function of a novel and facile luminescence sensor for PFOA based on iridium modified on gold surfaces. These surfaces were modified with lipophilic iridium complexes bearing alkyl chains, namely, IrC_6_ and IrC_12_, and Zonyl-FSA surfactant. Upon addition of PFOA, the modified surfaces **IrC**_**6**_**-FSA@Au** and **IrC**_**12**_**-FSA @Au** show the largest change in the red luminescence signal with changes in the luminescence lifetime that allow monitoring of PFOA concentrations in aqueous solutions. The platform was tested for the measurement of PFOA in aqueous samples spiked with known concentrations of PFOA and demonstrated the capacity to determine PFOA at concentrations >100 μg/L (240 nM).

## Introduction

Perfluoroalkyl substances (PFAS) possess desirable industrial characteristics such as oil and water repellency and good physical and chemical stability. They have been widely used in a range of applications: carpets, clothing, paper, and packaging to confer dirt, grease, and oil resistance as well as fire-fighting foams.^1^ However, in an environmental context, the strength of the C–F bond renders PFAS highly resistant to thermal, chemical, and biological degradation,^2^ with the result that they are capable of bioaccumulation and long-range environmental transport, exemplified by their presence in the Arctic.^3−5^ As a result and combined with concerns about toxicity (including the impaired response of children to vaccines),^6^ perfluorooctanoic acid (PFOA) and related PFOA compounds as well as perfluorooctanesulfonic acid (PFOS) and perfluorohexanesulfonic acid (PFHxS) are listed for elimination under the Stockholm Convention on Persistent Organic Pollutants (POPs).^7^ Additionally, long-chain analogues of PFOA, specifically C_9_–C_21_ perfluorocarboxylic acids (PFCAs), are under active consideration for listing under the Stockholm Convention. Moreover, the EU has listed PFOA and related PFAS as substances of very high concern,^8^ while the European Food Safety Authority (EFSA) has set a challenging tolerable weekly intake value of 4.4 ng/kg body weight for the sum of PFOA, PFOS, perfluorononanoic acid, and PFHxS;^9^ furthermore, as part of a recast of its Drinking Water Directive, the EU has set a limit of 100 ng/L for the sum of C_4_–C_13_ perfluorosulfonic acids and PFCAs (including PFOA) in drinking water.^10^ In March 2023, the United States Environmental Protection Agency (USEPA) announced proposed maximum contaminant levels (MCLs) for six PFAS in drinking water with that for PFOA set at 4 ng/L.^11^ To date, methods for the measurement of PFOA and related PFAS are based on gas or liquid chromatography interfaced with mass spectrometry (i.e., GC-MS and LC-MS).^12^ Despite being accurate and reproducible, such methods are time-consuming, require expert operators, are expensive, and cannot be carried out in the environment in real time. Instead, samples must be transported to a laboratory for analysis, with accompanying time delays. Against this backdrop, there is a clear and pressing need for a simple, rapid, and cost-effective method for measuring the concentrations of PFOA and related PFAS in aqueous samples. This need is particularly acute in situations such as real-time evaluation of the efficiency of treatment processes designed to remove PFAS from effluents discharged to surface water or in the case of chemical spills and first-response monitoring of PFAS concentrations in wastewater and groundwater, where the time requirements for laboratory analysis can result in serious environmental contamination in which concentrations of PFOA far exceed those detected in drinking water. Specifically, aqueous samples like industrial effluents and wastewater contain, e.g., up to 160 μg/L,^13^ while groundwater near military bases may contain up to 220 μg/L PFOA.^14^

It is challenging to design sensor systems for PFOA based on its chemical properties.^15^ Most sensor systems have relied on the interaction between organofluorines,^16,17^ which provide a recognition element for detection. Baker et al. estimated that the fluorine–fluorine (C–F···F–C) stabilization energy of up to 30 kcal/mol is adequate for perfluorinated molecules to be captured by another perfluorinated receptor.^18^ Luminescence is a very sensitive detection method, which can reach sensitivities down to the single molecule level. Luminescent sensors for the detection of PFOA have been developed using quantum dots,^19^ fluorescent organic dyes,^20,21^ gold nanoparticles,^22^ and metal–organic frameworks.^23,24^ While the photostability of the lumophore and its fluorescence quantum yield are important considerations in the designs, a key limitation in analytical detection arises from the mode of detection solely relying on luminescence intensity,^25,26^ which requires additional referencing controls and testing to exclude interferences with scattering, excitation light, or lumophore bleaching.

**Figure 1:**
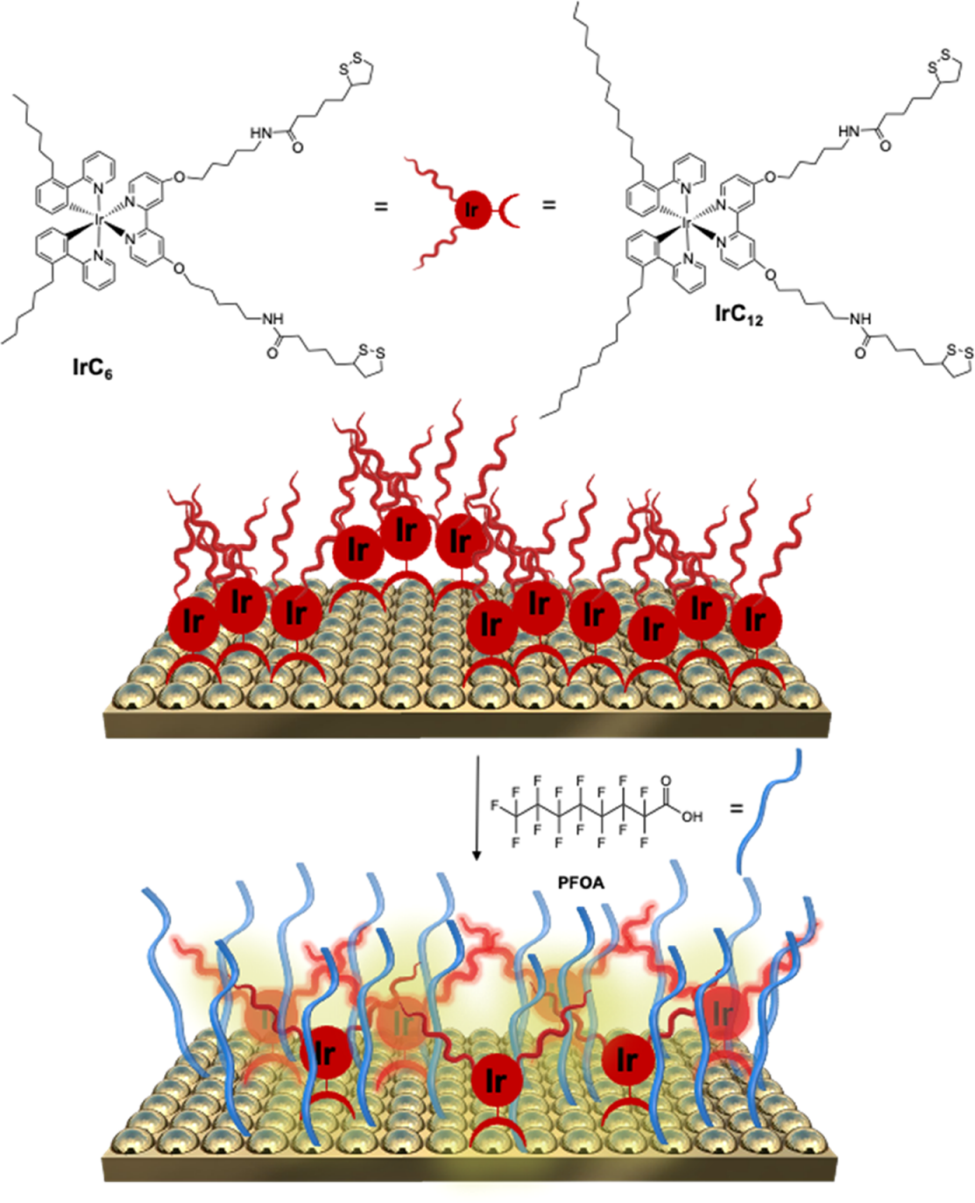
Schematic diagram of the functionalized gold surfaces, **IrC**_**6**_**@Au** and **IrC**_**12**_**@Au**, upon addition of PFOA. For clarity, micelle formation is indicated by aggregated complexes in the upper part, while a primary molecular surface coverage in the presence of the analyte is shown in the lower part to highlight the disassembly of the micelles.

Sensing systems based on the luminescence lifetime provide an attractive mode of detection with high sensitivity and versatility.^27^ Photostable lumophores with long luminescence lifetimes are ideal for usage in benchtop instruments for luminescence lifetime detection and have received wide popularity in medical diagnostics as luminescence lifetime detection is available in many microplate readers.^28^ Iridium-based luminescent probes have long-lived luminescence, in the range of hundreds of nanoseconds, in the visible region, originating from triplet charge transfer states. The iridium luminescence is responsive to the local microenvironment around the metal complex with changes that affect the rigidity of the complex, polarity, aggregation, and access of oxygen.^29−33^ We have previously anchored cyclometalated iridium complexes on gold solid supports using a thiol active ligand (bpySS), which prevented quenching from the gold surface, and studied the influence of the luminescence signal of the iridium films in the presence of proteins.^34−36^ Herein, we report the development of a novel solid-state luminescence lifetime-sensing platform for PFOA detection based on cyclometalated iridium complexes bearing lipophilic chains of 6 and 12 carbons, respectively, IrC_6_ and IrC_12_ (Figure 1). The hydrocarbon chains provide lipophilic tentacles on the iridium complex, ideal for inducing aggregates or organized self-assembled structures of the cationic iridium complexes. Agents such as PFOA which bear hydrophilic groups and lipophobic chains may interfere with the local iridium environment. We have examined the influence of PFOA on gold surfaces modified with two iridium complexes, **IrC**_**6**_**@Au** and **IrC**_**12**_**@Au**, using a series of surface characterization and analytical techniques. The luminescence lifetime of the iridium photoactive center is monitored under different conditions to optimize detection sensitivity and provide a monitoring tool for PFOA detection.

## Experimental Section

### Materials and Methods

Gold slides (30 nm on silicon with a 5 nm Ti priming layer) were purchased from Georg Albert PVD, Germany. The Zonyl-FSA fluorosurfactant and PFOA (95% purity) were purchased from Sigma-Aldrich. The PFOA native reference standard (1.2 mL, 50 μg/mL in methanol), the ^13^C_8_–PFOA internal (surrogate) standard (1.2 mL, 50 μg/mL in methanol), and the ^13^C_4_–PFOA recovery determination (syringe) standard (1.2 mL, 50 μg/mL in methanol) used for LC-MS analyses were purchased from Wellington Laboratories, Canada. The syntheses and characterization of IrC_6_, IrC_12_, and IrC_12_bpy complexes are described in Supporting Information (SI) Part 1.1. The complexes Ir(ppy)_2_bpy and IrbpySS are prepared as previously reported.^29^ The rest of the chemical agents and solvents used in this study were obtained from Sigma-Aldrich, Fluka, Fisher Scientific, or Acros Chemicals and used without further purification. Phosphate buffer solutions (pH = 7.4) were prepared by mixing 0.07 M stock solutions of sodium phosphate dibasic dihydrate (Na_2_HPO_4_·2H_2_O) and potassium phosphate monobasic (KH_2_PO_4_) in 7:3 (v/v) ratio.

**IrC**_**6**_**@Au** and **IrC**_**12**_**@Au**: Gold slides were rinsed thoroughly with acetonitrile and milli-Q water sequentially three times before immersion in 80 °C piranha solution (concentrated sulfuric acid and hydrogen peroxide in a proportion of 3:1 (v/v)) for 15 min to eliminate all organic residues on the surface. The cleaned surfaces were subsequently washed with milli-Q water, dried with a stream of nitrogen, and stored in ethanol until use. The **IrC**_**6**_**@Au** and **IrC**_**12**_**@Au** surfaces were prepared by immersing the gold slides in 0.74 μM IrC_**6**_ acetonitrile solution or 0.62 μM IrC_12_ acetonitrile solution for 18 h. The **IrC**_**6**_**-FSA@Au** and **IrC**_**12**_**-FSA@Au** surfaces were prepared by immersion in a 0.74 μM IrC_6_ acetonitrile solution with 2% Zonyl-FSA or a 0.62 μM IrC_12_ acetonitrile solution with 2% Zonyl-FSA for 18 h, in a final solvent composition of 92% MeCN, 4% water, and 4% isopropyl alcohol. All modified surfaces were washed with small amounts of acetonitrile after immersion and dried with a stream of nitrogen. The prepared surfaces were finally stored in a dark environment under a nitrogen atmosphere until use. For the detection of PFOA, the modified surfaces were immersed in phosphate-buffered solutions (pH of 7.4) with PFOA for 30 min of incubation and subsequently rinsed with acetonitrile and dried with a stream of nitrogen. The functionalized surfaces were analyzed using two systems of scanning electron microscopy (SEM): (i) a CFEI Quanta 3D FEG FIB-SEM fitted with an Oxford Inca 300 energy dispersive X-ray spectrometer (EDS) at the University of Birmingham and (ii) a Zeiss Supra 40 SEM with a Schottky field emitter having attached a Thermo Fisher Scientific EDS (Waltham, MA, USA) at BAM. Dynamic light scattering (DLS) was carried out using a Malvern Zetasizer nano-ZSP. Steady-state and time-resolved luminescence studies were conducted on an Edinburgh Instruments FLS920 spectrophotometer fitted with a 450 W xenon arc lamp as an excitation source and a Hamamatsu R928 photomultiplier tube as a detection system. The data were collected with F980 software and corrected for photomultiplier tube and instrument responses. Lifetime spectra were obtained with an EPL-375 laser as an excitation source and were fitted with FAST software, with an estimated error of 10% and χ^2^ within 1 ± 0.2. Semiquantitative determination of the surface chemical composition was carried out using time-of-flight secondary ion mass spectrometry (TOF-SIMS) with a ToF-SIMS IV instrument (IONTOF, Münster, Germany). X-ray photoelectron spectroscopy (XPS) studies were performed using a Thermo NEXSA XPS fitted with a monochromated Al kα X-ray source (1486.7 eV), a spherical sector analyzer, and 3 multichannel resistive plate, and 128 channel delay line detectors. Ultraviolet–visible (UV–vis) absorption spectra were recorded on a Cary 60 UV–vis spectrophotometer.

#### LC-MS Methods for the Determination of PFOA in Water

LC-MS determination of concentrations of PFOA in water samples was conducted using a Sciex Exion HPLC coupled to a Sciex 5600+ triple TOF MS instrument equipped with a Restek Raptor C18 column (1.8 μm particle size, 50 mm length, 2.1 mm internal diameter). PFOA extraction was carried out using Phenomenex Strata TM-X-AW 33 μm polymeric weak anion, 200 mg/6 mL solid-phase extraction (SPE) cartridges. Further experimental details about the solid-phase extraction of PFOA and LC-MS instrumental settings are provided in SI Parts 1.2 and 1.3. Specifically, the liquid chromatography elution program and the MS/MS transitions employed are shown in Tables S2 and S3. Relative standard deviation = 34%.

### Sample Collection and Treatment

29 samples were prepared for evaluation of the sensor. These comprised bottled water (eight still mineral waters, six sparkling waters, and five flavored water samples) purchased from supermarkets, along with tap water sampled from 10 different household kitchens in Birmingham, U.K. The water samples were collected between October and November 2020. For quality control, blank samples were prepared in the guise of distilled deionized (Milli-Q) purified laboratory reagent water collected in a PET sample bottle. Bottled water samples (different brands/water sources/types) were purchased from four different shops in Birmingham in October 2020. All samples were transferred promptly to the laboratory and stored at 4 °C before analysis.

The water samples were first analyzed by LC-TOF-MS to measure the concentration of PFOA before detection by the iridium-functionalized surfaces. To evaluate the performance of the prepared sensor, the same water samples were then spiked with known concentrations of PFOA within the range detectable by the sensor (herein, 10 and 100 μg/L).

## Results and Discussion

### Characterization of Ir(III)-Functionalized Surfaces

**Figure 2:**
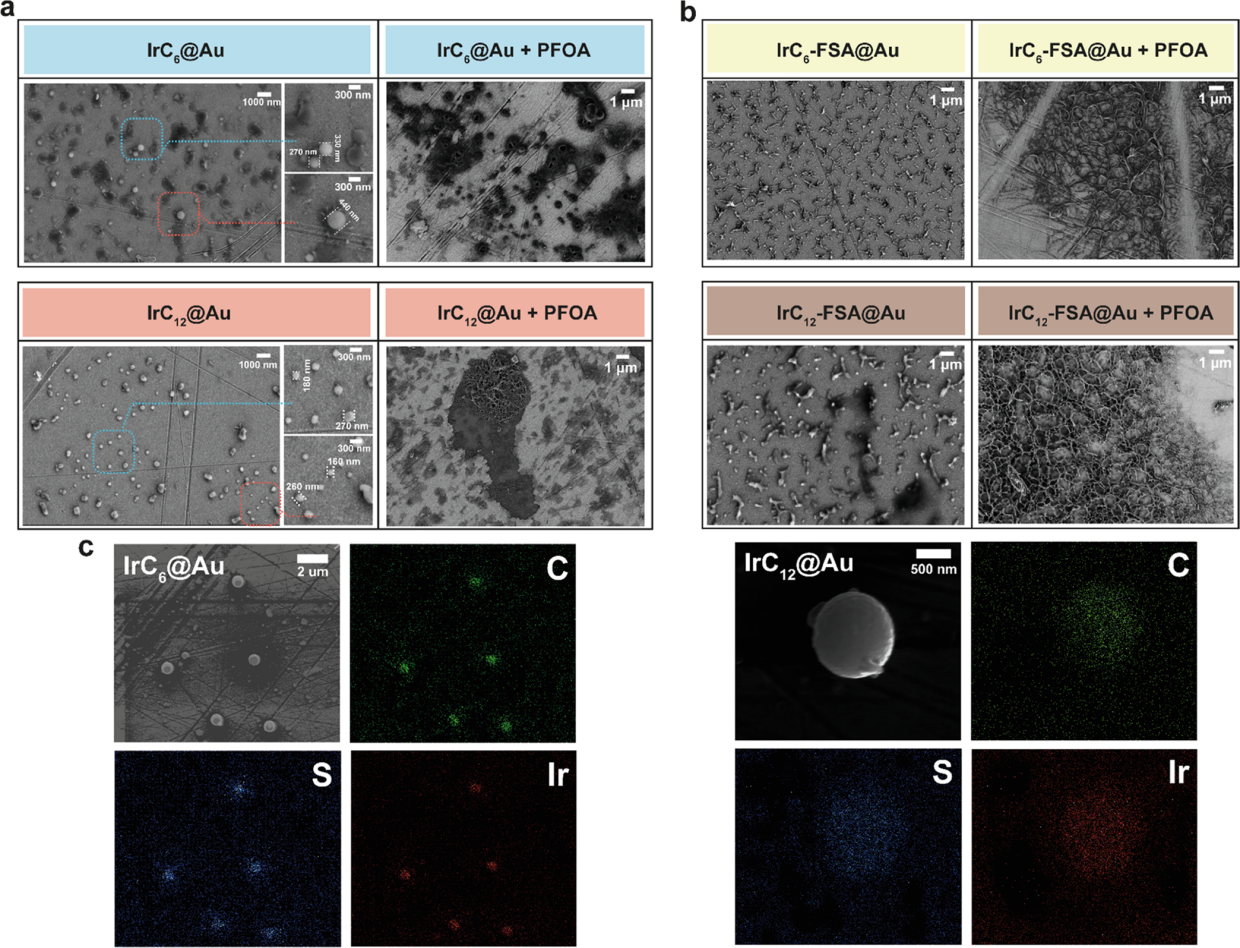
(a) SEM images of **IrC**_**6**_**@Au** and **IrC12@Au** without and with PFOA and (b) **IrC**_**6**_**-FSA@Au** and **IrC**_**12**_**-FSA@Au** without and with PFOA. Solution of PFOA: 1 g/L for **IrC**_**6**_**@Au** and **IrC**_**12**_**@Au** and 10 mg/L for **IrC**_**6**_**-FSA@Au** and **IrC**_**12**_**-FSA@Au**. (c) EDS elemental mapping analysis (carbon, sulfur, and iridium) for **IrC**_**6**_**@Au** and **IrC**_**12**_**@Au**.

The luminescent iridium probes, IrC_6_ and IrC_12_, were designed with surface-active dithiol groups for covalent attachment to gold surfaces. The surfaces have been fully characterized using analytical techniques to examine the morphology and elemental constitution. First, SEM was used to characterize the morphology of the modified surfaces **IrC**_**6**_**@Au** and **IrC**_**12**_**@Au** (Figure 2a). Interestingly, both IrC_6_ and IrC_12_ show the formation of well-dispersed micelle-type structures on the Au surface. Their mean sizes were found to be 250 ± 40 nm in **IrC**_**6**_**@Au** and 230 ± 30 nm in **IrC**_**12**_**@Au** (Figure S2a,b). Upon investigation of the analogous iridium complexes without the lipophilic 6 and 12 carbon chains (Ir(ppy)_2_bpy and IrbpySS), carrying either dithiol chains as an anchor or not, no micelles were observed on the surfaces (Figure S1). EDS elemental mapping analysis of the **IrC**_**6**_**@Au** and **IrC**_**12**_**@Au** (Figure 2c) shows the clear presence of Ir and organic contents on the micelle-type structures. Independent DLS analysis of acetonitrile solutions of IrC_6_ and IrC_12_ confirmed the presence of micelles in solution, with sizes of 210 ± 6 and 190 ± 3 nm for IrC_6_ and IrC_12_, respectively, which are in the same range but slightly smaller than the ones observed on gold surfaces (Table S1). The smaller size of the IrC_12_ micelles can be attributed to more efficient packing of the aliphatic chains; hence, smaller size micelles are observed. This is supported by independent examination of surfaces coated with the iridium complexes under the same conditions with low iridium concentrations (0.15 μM); in this case, no micelles were observed on the surfaces, supporting the estimated concentration required for micelle formation on the surface. Upon addition of PFOA, the morphology of the **IrC**_**6**_**@Au** and **IrC**_**12**_**@Au** surfaces was examined. A significant disruption of the micelle-type structures can be observed (Figure 2a). It is postulated that the hydrophobic and oleophobic fluorosurfactant PFOA interacts with the hydrophobic micelle interior, leading to micelle disassembly. The coating of the surfaces with iridium was also examined in the presence of a fluorinated surfactant, Zonyl-FSA, which was previously used with iridium surface-active complexes on gold surfaces,^28,29^ as its fluorinated chain was envisaged to affect the PFOA interaction. The **IrC**_**6**_**-FSA@Au** and **IrC**_**12**_**-FSA@Au** surfaces show iridium aggregation with elongated shape structures around micelle-type features (Figure 2b), and upon EDS analysis, it was found that the elemental composition of fluorine was 32 atom % (Figure S2c). Addition of PFOA led to the disruption of the structures (Figure 2b) and EDS analysis reveals a lower fluorine content (20% atom) possibly due to loss of the Zonyl-FSA (Figure S2c).

Contact angle measurements of gold surfaces coated with IrC_6_ and IrC_12_ were carried out to probe the effect of chain length on the relative hydrophobicity of the surfaces. The trend of the measurements (Figure S3) from 60° of the plain gold surface to 73° **IrC**_**6**_**@Au** and 80° **IrC**_**12**_**@Au** shows that modification of the surface with the iridium complexes led to an increase of surface hydrophobicity.

**Figure 3:**
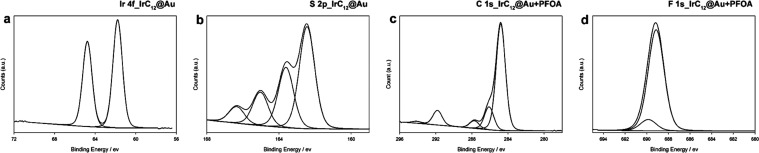
XPS spectra of (a) Ir 4f spectrum of **IrC**_**12**_**@Au**, (b) S 2p spectrum of **IrC**_**12**_**@Au**, (c) C 1s spectrum of **IrC**_**12**_**@Au** with PFOA (PFOA concentration: 100 mg/L), and (d) F 1s spectrum of **IrC**_**12**_**@Au** with PFOA (PFOA concentration: 100 mg/L).

X-ray photoelectron spectroscopy (XPS) was used to further characterize the modified surfaces. The characteristic Ir 4f and S 2p peaks confirm the attachment of the complexes to the surfaces. The characteristic peaks are similar for **IrC**_**6**_**@Au** and **IrC**_**12**_**@Au**; hence, only selected peaks for **IrC**_**12**_**@Au** are shown in Figure 3 and the peaks for **IrC**_**6**_**@Au** are shown in Figure S4. The peaks for Ir (Figure 3a) appeared at 61.8 eV (4f_7/2_) and at 64.8 eV Ir (4f_5/2_) in all modified surfaces The binding energy and the symmetric peak shape confirm the ionic character of the Ir.^30^ The S 2p region for **IrC**_**6**_**@Au** and **IrC**_**12**_**@Au** (Figure 3b) revealed peaks at 162.5 eV and 163.7 eV, (characteristic of 2p_3/2_ peaks) assigned to thiolate (48%) and disulfide (28%), respectively.^31^ The two additional peaks at 165.2 (16%) and 166.8 eV (8%) are attributed to oxidized sulfur as previously reported. The presence of disulfide may be attributed to some of the surface-active groups not attached to the surface, with some Ir(III) complexes binding only with one thiolate-capped leg. The remaining oxidized species are usually regarded as sulfonate as previously observed.^32^ Furthermore, upon addition of PFOA, the peaks of CF_2_ (291.8 eV) and CF_3_ (294.2 eV) appear clearly in the region of the carbon peaks (Figure 3c) and the F peaks are shown with addition of PFOA (Figure 3d).

### Probing Immobilization of PFOA and Zonyl-FSA on Coated Surfaces

The immobilization of PFOA or Zonyl-FSA on coated surfaces was measured by TOF-SIMS, which is a highly surface-sensitive method capable of analyzing the top 1–3 nm of a substrate.^33^ The primary ion beam used for ToF-SIMS analysis causes molecular fragmentation of the analyte, and certain species can be identified by their characteristic ions. Because the yield of secondary ions is dependent on several parameters including the surrounding matrix, ToF-SIMS studies may draw conclusions from the presence or absence of peaks as well as large variations (orders of magnitude) in peak intensities; however, a fully quantitative study is possible only under set conditions.^34,35^ The correct interpretation of peak intensities in this study relies on suitable scaling of the *y*-axis. Here, all peaks were scaled to the area of the C_3_H_7_NO^+^ peak from the amide group in the IrC_12_ complex, which is common to all samples and was present in a suitably high intensity to give reliable scaling. Other peaks such as those related to Ir did not provide a high enough signal intensity to be suitable as a reference peak. It is also demonstrated from the EDS atomic composition results that the same amount of the iridium complex is deposited on the different surfaces.

**Figure 4:**
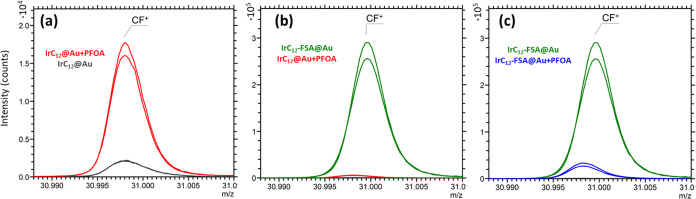
TOF-SIMS spectra showing the characteristic CF^+^ peak for the presence of PFOA and Zonyl-FSA in comparisons of substrates: (a) **IrC**_**12**_**@Au** (black) and **IrC**_**12**_**@Au** + PFOA (red), (b) **IrC**_**12**_**-FSA@Au** (green) and **IrC**_**12**_**@Au** + PFOA (red), and (c) **IrC**_**12**_**-FSA@Au** (green) and **IrC**_**12**_**-FSA@Au** + PFOA (blue). Spectra were normalized to the C_3_H_7_NO^+^ peak at *m*/*z* = 73.05 present in the amide group in the IrC_12_ complex. Samples were analyzed in duplicate, with repeats shown as separate lines.

The CF^+^ secondary ion is readily formed from fluorocarbons and is common to both Zonyl-FSA and PFOA and is therefore useful for a comparison of the relative adsorption of PFOA or Zonyl-FSA on the substrate (Figure 4). A comparison of the **IrC**_**12**_**@Au** substrate before and after PFOA treatment indicates some immobilization of PFOA on the substrate (Figure 4a). The weak CF^+^ signal for the unexposed substrate **IrC**_**12**_**@Au** may be caused by residual CF species in the ToF-SIMS instrument or alternatively from cross-contamination between samples. A comparison of the substrates **IrC**_**12**_**@Au**+PFOA and **IrC**_**12**_**-FSA@Au** shows an order of magnitude change in the CF^+^ peak, indicating that Zonyl-FSA has extensive adsorption on the substrate. Interestingly, the **IrC**_**12**_**-FSA@Au** substrate shows a significant drop in the intensity of the CF^+^ peak, suggesting that PFOA interferes with the immobilization of Zonyl-FSA. Comparisons of peak areas are shown in Figure S5 for both CF^+^ and Li^+^ peaks. The Li^+^ peak is an independent indicator of Zonyl-FSA. The significantly higher peak area for the **IrC**_**12**_**-FSA@Au** compared **to IrC**_**12**_**-FSA@Au**+PFOA supports the conclusions drawn from the CF^+^ peak that PFOA has affected the Zonyl-FSA immobilization. These results are supported by EDS analysis and further examined by luminescence lifetime measurements below.

### Detection of PFOA by Iridium Luminescence

**Figure 5:**
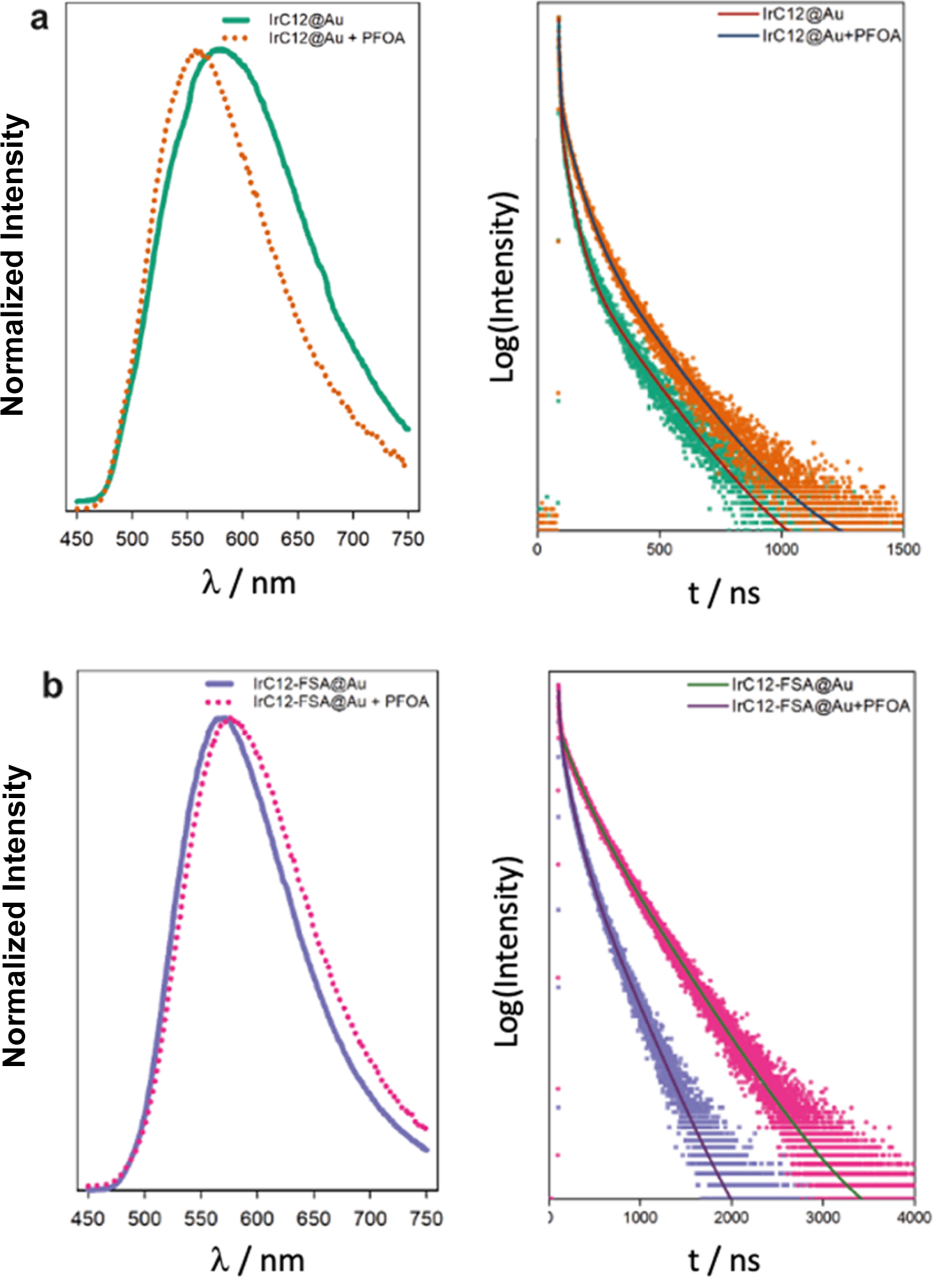
Selected luminescence spectra and normalized lifetime decays (fit-solid line) upon addition of PFOA to (a) **IrC**_**12**_**@Au** ([PFOA] = 1g/L) and (b) **IrC**_**12**_**-FSA@Au** ([PFOA] = 10 mg/L).

The luminescence properties of the iridium micelles on surfaces were characterized by steady-state and time-resolved spectroscopy and compared with the complexes in solution before and after addition of PFOA and in the presence of the FSA surfactant. The iridium-modified surfaces, **IrC**_**6**_**@Au** and **IrC**_**12**_**@Au**, exhibit characteristic luminescence originating from the triplet charge transfer state with maxima at 570 and 574 nm upon excitation at 375 nm (Table 1 and Figure 5). The luminescence signal is significantly blue-shifted compared to the acetonitrile solutions of the complexes at 608 and 611 nm (Table S4). Interestingly, the luminescence lifetime originating from the iridium complexes on the surface (Table 1) is significantly longer (the major component of 150 and 165 ns for **IrC**_**6**_**@Au** and **IrC**_**12**_**@Au**, respectively) than the corresponding solutions of the complexes in acetonitrile (55 and 62 ns for **IrC**_**6**_ and **IrC**_**12**_, respectively, Table S4), which is unusual as quenching of metal complex luminescence by gold surfaces has been commonly observed.^37,38^ It is worth noting that the multicomponent luminescence lifetime is commonly observed for iridium complexes based on the mixed character of the triplet excited state, and we focus the comparisons on the longer, major component. We attribute the longer lifetimes to the surface arrangement of the complexes. Exposure of the **IrC**_**6**_**@Au** and **IrC**_**12**_**@Au surfaces** to PFOA leads to a significant further blue shift of the signal at 560 nm, accompanied by an increase of the luminescence lifetime of 31 ns for **IrC**_**6**_**@Au** and 26 ns for **IrC**_**12**_**@Au** (Figure 5). The addition of PFOA in the solutions of **IrC**_**6**_ and **IrC**_**12**_ did not show any changes of the maximum of the emission intensity with a small increase of the luminescence lifetimes (Table S4). The effect of PFOA on the Ir-modified surfaces may be attributed to the disassembly of the micellar structures as also observed by SEM, which can be driven by association of the PFOA with the iridium complex leading to a number of factors that influence the luminescence lifetime such as protection from luminescence quenching by ^3^O_2_ or change in the polarity of the environment around the metal center due to the disruption of the micellar structure. We examined the iridium surfaces cocoated with the Zonyl-FSA surfactant, which was previously shown to lead to lengthening of the luminescence lifetime of metal complexes.^35^ It was envisaged that the presence of the surfactant on the surface (as confirmed by surface analysis) enhances the interaction of PFOA with the surface due to the presence of its fluorinated chain. The surfaces coated with the fluorinated surfactant, **IrC**_**6**_**-FSA@Au** and **IrC**_**12**_**-FSA@Au**, exhibit long luminescence lifetimes (446 and 502 ns, respectively), as anticipated, while the micellar structures are maintained with elongated assemblies evidenced by SEM (Figure 2). Upon addition of PFOA, the luminescence lifetimes are decreased by 139 ns for **IrC**_**6**_**-FSA@Au** and by 194 ns for **IrC**_**12**_**-FSA@Au**, which are much larger changes than the ones observed with the **IrC**_**16**_**@Au** and **IrC**_**12**_**@Au** surfaces. These changes are attributed to the replacement of the Zonyl-FSA by PFOA in the iridium microenvironment accompanied by changes of the surface assemblies. The replacement of Zonyl-FSA is also supported by the aforementioned results of the EDS and TOF-SIMS analyses, which confirm the change of the fluorine content on the surfaces.

**Table 1:** Emission Maxima and Luminescence Lifetimes of Iridium-Modified Gold Surfaces upon the Addition of PFOA^a^

name	λ_em_ (nm)	τ (ns)	
IrC_6_@Au	570	13 ± 1 (21%)	150 ± 4 (79%)
IrC_6_@Au + PFOA	561	49 ± 3 (24%)	191 ± 2 (76%)
IrC_6_–FSA@Au	562	119 ± 6 (19%)	446 ± 5 (81%)
IrC_6_–FSA@Au + PFOA	571	76 ± 1 (25%)	307 ± 7 (75%)
IrC_12_@Au	574	32 ± 3 (26%)	165 ± 2 (74%)
IrC_12_@Au + PFOA	560	54 ± 2 (23%)	194 ± 3 (77%)
IrC_12_–FSA@Au	569	167 ± 5 (20%)	502 ± 4 (80%)
IrC_12_–FSA@Au + PFOA	578	93 ± 7 (25%)	308 ± 3 (75%)

a PFOA solutions added: 1 g/L for **IrC_6_@Au** and **IrC_12_@Au** and 10 mg/L for **IrC_6_-FSA@Au**, **IrC_12_-FSA@Au**. The amplitude of each lifetime component is shown in brackets. Estimated errors: λ_em_ ± 1 nm and luminescence lifetime error with standard deviation (*n* = 3).

### Analytical Performance and Optimization for PFOA Detection

**Figure 6:**
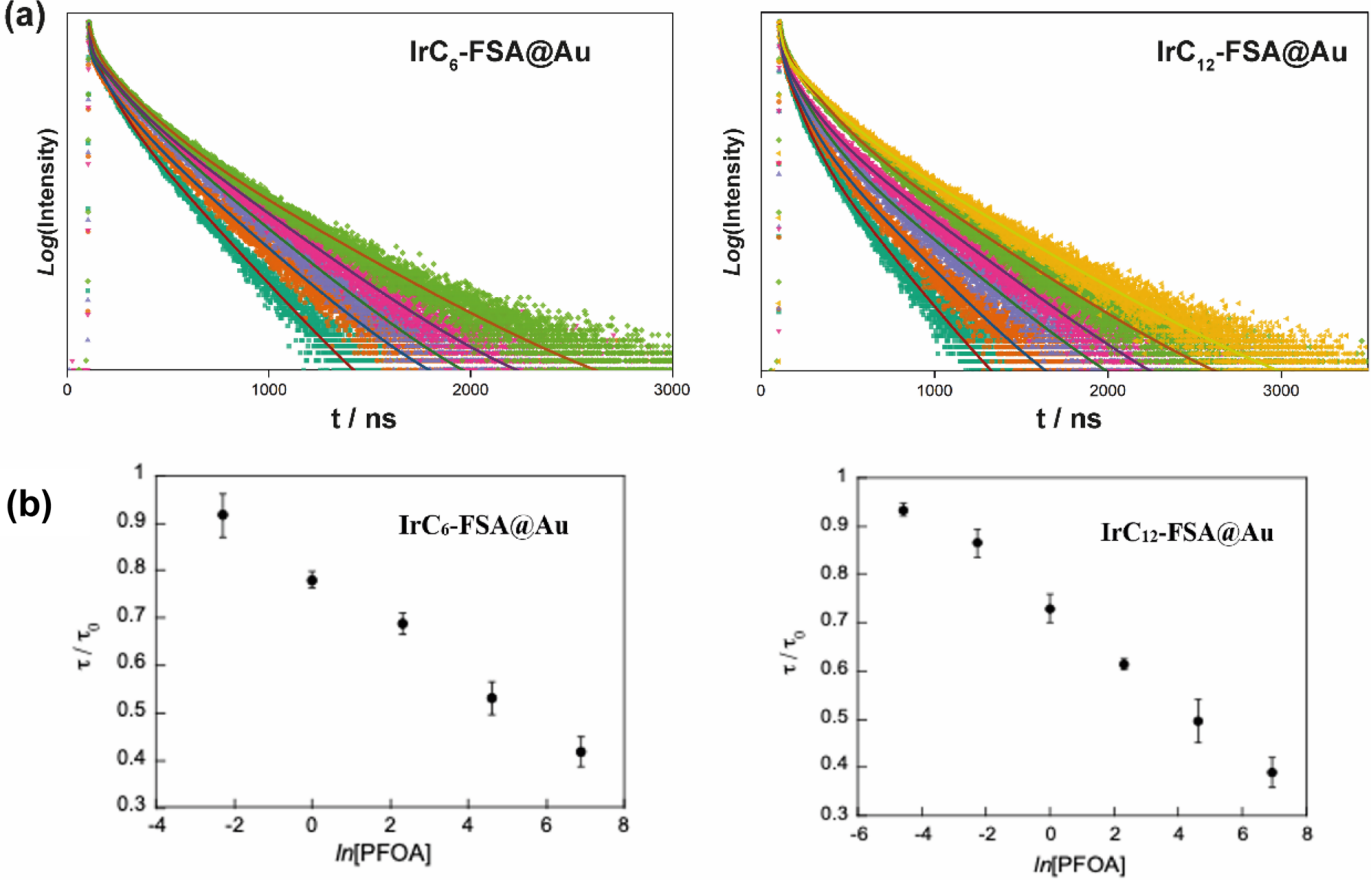
Effect of addition of PFOA on luminescence lifetime: (a) luminescence lifetime decays normalized (fit -solid line) and (b) plot of τ/τ_0_ against ln[PFOA] with [PFOA] in mg/L, a pH of 7.38 (range of 100 μg/L–1 g/L for **IrC**_**6**_**-FSA@Au** and 10 μg/L–1 g/L for **IrC**_**12**_**-FSA@Au**).

To examine the sensitivity and range of the platform, the luminescence lifetime was monitored across a range of PFOA concentrations for the surfaces with (**IrC**_**6**_**-FSA@Au** and IrC_**12**_**-FSA@Au**, Figure 6) and without (**IrC**_**6**_**@Au** and **IrC**_**12**_**@Au**, Figure S7) the Zonyl-FSA surfactant. All surfaces show an exponential dependence of the luminescence lifetime upon the addition of PFOA (Figures 6 and S7). If the PFOA interaction was based on a bimolecular event between the PFOA and the Ir(III) complex, a linear dependence would have been expected between the luminescence lifetime and the concentration of PFOA. However, the exponential dependence with the concentration of PFOA is consistent with lumophore sensing within micelles.^39,40^ It is expected that the PFOA will interact with the lumophores within the micelle according to previously studied models.^41,42^ The shifts of the emission maxima observed also agree with a ground-state association of PFOA within the micelles on the surface, which indicates that the effect of the luminescence lifetime may also be attributed to static quenching based on the extent of PFOA association. Nevertheless, this dependence correlates well with the luminescence lifetime and allows the estimation of concentration. The limit of detection (LOD) was estimated to be 8.2 mg/L (20 μM, S/N = 3) for IrC_6_@Au + PFOA and 67 mg/L (162 μM, S/N = 3) for the IrC_12_@Au surface. The cocoated surfaces showed a higher LOD as expected from the initial screening of luminescence properties with a LOD for IrC_12_–FSA@Au + PFOA of 39 μg/L (94 nM, S/N = 3) and detection range from 100 μg/L (0.24 μM) to 1 g/L (2.42 mM); the IrC_12_–FSA@Au + PFOA surface has a LOD of 6.2 μg/L (15 nM, S/N = 3) and detection range from 10 μg/L (24.2 nM) to 1g/L (2.42 mM) (Table S5).

The surfaces show reproducible performance with standard deviations as indicated in the luminescence lifetime tables. The surface reproducibility was examined with 50 independent measurements of the luminescence lifetime of iridium-coated surfaces.

**Figure 7:**
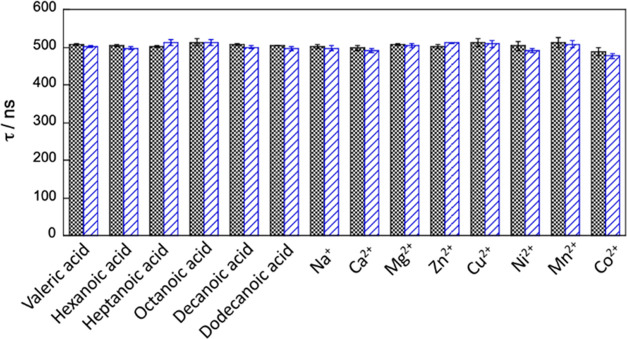
Influence of the possible interferents Na^+^, Ca^2+^, Mg^2+^ Zn^2+^, Cu^2+^, Ni^2+^, Mn^2+^, and Co^2+^ and valeric acid, hexanoic acid, heptanoic acid, octanoic acid, decanoic acid, and dodecanoic acid (1 mg/L) on the luminescence lifetime of iridium, upon addition of the interferent to the IrC_12_–FSA@Au surface; before (dark) and after (light).

We also examined the possible interference of metal cations and other acids, which are reported as common interferents in a large number of luminescence sensors.^36^ The following cations and aliphatic acids were studied for possible interference on the luminescence of **IrC**_**12**_**-FSA@Au:** Na^+^, Ca^2+^, Mg^2+^ Zn^2+^, Cu^2+^, Ni^2+^, Mn^2+^, and Co^2+^ and valeric acid, hexanoic acid, heptanoic acid, octanoic acid, decanoic acid, and dodecanoic acid. The luminescence lifetimes of **IrC**_**12**_**-FSA@Au** before and after the addition of the possible interferents are shown in Figure 7. The variation (−2–2%) of the luminescence lifetime is very small and within the experimental error margins. In conclusion, both the selected metal ions and the aliphatic acids did not display any interferences with the platform’s luminescence signal.

Overall, the performance of the iridium-modified surfaces shows strong potential for further development of the luminescence lifetime as a detection technique for PFOA. Analysis of polluted wastewater effluents has revealed concentrations of PFOA of 160 μg/L.^13^ It is challenging to compare the different detection approaches for the development of novel sensors as many factors need to be taken into consideration apart from sensitivity of detection: interferences, time for response, detection versatility, and stability. Optical techniques offer a great advantage of rapid detection compared to electrochemical sensors, although the latter have reported high sensitivities.^43,44^ Most of the reported platforms require incubation time between 20 and 30 min, which compare well with the iridium surface platform, although some methods require preconcentration steps or longer incubation periods.^45,46^ The iridium platform also provides great stability of the chromophore compared to organic counterparts and has strong potential in development of a portable device.^47^

### Drinking Water Analysis

To evaluate the feasibility of the sensing platform, **IrC**_**12**_**-FSA@Au** was applied in the analysis of 29 samples of drinking water from the UK West Midlands. These samples were first analyzed for their PFOA content using LC-TOF-MS before being analyzed by our sensor. As shown in Table 2, concentrations of PFOA in unspiked bottled water ranged between 0.67 and 3.9 ng/L, while those in tap water were in the range of 0.82–1.4 ng/L. These concentrations were well within the EU Drinking Water Directive limit (100 ng/L for the sum of C_4_–C_13_ PFCAs and PFSAs) but were in some instances close to exceeding the MCL for PFOA announced recently by the USEPA of 4 ng/L.^11^ Compared with previously reported data (Table S6), PFOA concentrations in our UK samples are very similar to those reported in Ireland, Norway, Germany, Belgium, and the Netherlands but are exceeded by those reported in Italy (northern Milan), Spain (Catalonia), the USA (24 contiguous states), and China (79 cities). Given that the concentrations of PFOA in the U.K. drinking water samples are below the detection limit of our sensor, samples were instead spiked with the PFOA reference standard at two concentrations. Tap water was spiked at 10 mg/L and at 100 μg/L, and these were measured using the sensor. In the 10 mg/L spiked samples, PFOA was detected between 7 and 14 mg/L, while in the 100 μg/L spiked samples, PFOA was detected in the range of 88–151 μg/L. These results suggest that in aqueous samples like industrial effluents and wastewater that contain, e.g., up to 160 μg/L^13^ or groundwater near the military base that contains up to 220 μg/L,^14^ this luminescence sensor is applicable and reliable. We believe that further optimization of our approach will yield lower detection limits and thus widen its applicability. This Ir(III) luminescent sensor has strong potential to overcome issues with the cost and time-consuming nature of LC-MS-based methods for PFOA/PFAS detection, which limit their widespread large-scale use.

**Table 2:** Concentrations of PFOA Detected in Drinking Water Unspiked^a^ and Spiked^b^ at 100 and 10 mg/L

	PFOA concentration		
water type	unspiked tap water (ng/L)	tap water (spiked at 10 mg/L) (mg/L)	tap water (spiked at 100 μg/L) (μg/L)
mineral water 1	0.68	12	120
mineral water 2	0.71	12	89
mineral water 3	0.69	13	109
mineral water 4	0.72	11	112
mineral water 5	0.71	9	97
mineral water 6	0.72	12	134
mineral water 7	0.83	12	137
mineral water 8	0.73	14	120
sparkling water 1	0.72	11	88
sparkling water 2	0.76	11	110
sparkling water 3	0.74	14	88
sparkling water 4	0.72	9	94
sparkling water 5	0.71	11	101
sparkling water 6	0.67	7	88
flavored water 1	0.67	13	126
flavored water 2	3.9	11	116
flavored water 3	1.7	8	96
flavored water 4	1.0	10	142
flavored water 5	2.2	14	105
tap water 1	0.87	11	102
tap water 2	0.98	8	127
tap water 3	0.90	9	151
tap water 4	1.0	9	114
tap water 5	0.94	13	111
tap water 6	0.96	8	124
tap water 7	0.97	9	114
tap water 8	0.90	9	127
tap water 9	1.4	12	136
tap water 10	0.82	13	93

a Determined using LC-TOF-MS.

b Determined using the IrC12-FSA@Au sensor.

## Conclusions

We have demonstrated that gold surfaces modified with IrC_6_ and IrC_12_ metal probes provide a stable, reliable, and sensitive optical platform for the detection of PFOA based on the iridium luminescence lifetime signal for the rapid detection of PFOA in aqueous media at concentrations down to 100 μg/L and in the presence of a range of interferents. The iridium complexes have lipophilic chains, which affect the assembly on gold with evidence of micellar-type structures. Addition of PFOA affects the luminescence lifetime of the iridium probes, which are known for sensitivity of the luminescence signal on the local environment. Characterization analysis of the surfaces shows that addition of PFOA disrupts the micellar-type assemblies on the surfaces. The change of the luminescence lifetime in the presence of PFOA is attributed to the interaction of PFOA with the iridium assemblies on the surface. The largest change of the lifetime, which is best suited for analytical detection, is observed for the gold surfaces cocoated with the fluorinated surfactant Zonyl-FSA and IrC_6_ or IrC_12_. The Zonyl-FSA surfactant not only enhances the PFOA interaction with the iridium-coated surfaces but by increasing the luminescence lifetime also provides a large, analytically well-exploitable change of the lifetime signal upon displacement with PFOA. The sensory surfaces effectively quantify PFOA concentrations in drinking water down to 100 μg/L (240 nM) and display extraordinary anti-interference. In summary, the surface-based luminescence assays reported here provide a novel approach to monitoring PFOA and related PFAS based on the iridium probe luminescence lifetime signal with rapid screening and a large window of detection, which can be further exploited for the development of multianalyte devices. The approach shows the strong potential of the luminescence lifetime and iridium-based sensors for the development of devices for the detection of environmental pollutants.

## Supporting Information Available

Synthetic experimental methods and NMR spectra; solid-phase extraction method for PFOA from water samples; LC-TOF-MS instrument parameter settings; SEM images of control surfaces Ir(ppy)2Bpy, IrBpySS, and IrC12Bpy, along with dynamic light scattering measurements; XPS spectra of IrC6@Au and EDX spectra; contact angle measurements, and photoluminescence lifetimes of surfaces to varying PFOA concentration and concentrations of PFOA in drinking water across different countries (PDF)
