# Supplementary material for: Luminescence Lifetime-Based Sensing Platform Based on Cyclometalated Iridium(III) Complexes for the Detection of Perfluorooctanoic Acid in Aqueous Samples
Source: Anal Chem. 2024 Jan 16;96(4):1565–75. doi: 10.1021/acs.analchem.3c04289 (PMC10831797; doi:10.1021/acs.analchem.3c04289)
Supplement: Supplementary file 1 — ac3c04289_si_001.pdf [file ac3c04289_si_001.pdf]

Supporting Information

Luminescence Lifetime-Based Sensing Platform Based on Cyclometalated Iridium (III) Complexes for the Detection of Perfluorooctanoic Acid in Aqueous Samples

Kun Zhang, [#] [a,b] Andrew J. Carrod, [#, a,e] Elena Del Giorgio,[a] Joseph Hughes,[a] Knut Rurack,[c] Francesca Bennet,[d,f] Vasile-Dan Hodoroaba,[d] Stuart Harrad,[b*] and Zoe Pikramenou[a*]

[a]School of Chemistry, University of Birmingham, Birmingham, B15 2TT, U.K

[b]School of Geography, Earth & Environmental Sciences, University of Birmingham, Birmingham, B15 2TT, U.K.

[c]Chemical and Optical Sensing Division, Federal Institute for Materials Research and Testing (BAM), Richard-Willstätter-Str. 11, 12489 Berlin, Germany

[d]Surface Analysis and Interfacial Chemistry Division, Federal Institute for Materials Research and Testing (BAM), Unter den Eichen 44-46, 12203 Berlin, Germany

[e] Current address: Department of Chemistry and Molecular Biology, University of Gothenburg, Gothenburg 41296, Sweden

[f] Current address: Unit Product Analytics, Department of Chemicals and Product Safety, Federal Institute for Risk Assessment, Max-Dohrn-Strasse 8-10, 10589 Berlin, Germany

joint first authors[#]
*corresponding authors: s.j.harrad@bham.ac.uk; z.pikramenou@bham.ac.uk

Contents

1. Further experimental details 2
 1.1 PFOA Solid-phase extraction 2
 1.2 LC-TOF-MS setting 2
2. Tables and Figures 3
 2.1 Surface characterisation 3
 2.2 LC-MS analysis 5
 2.3 Photophysical analysis 5
3. Syntheses of Ir (III) Complexes 8
  BpySS 8
  IrC_6 9
  IrC_{12} 13
  $IrC_{12}bpy$ 18
4. Additional references 20

1. Further experimental details

1.1 PFOA Solid-phase extraction

To concentrate PFOA, this study adopted an approach that has previously been reported by our group.[3] Briefly, 50 μL of 1 ng/μL internal standard (M8-PFOA) dissolved in methanol was first added in the 500 mL filtered water, subsequently pass them through a 0.45 μm membrane for removing impurities. The solid-phase extraction (SPE) cartridges were conditioned with 2×6 mL of 0.1% NH_4OH/methanol and 6 mL Milli-Q water in advance before extracting the spiked water samples at around 1-2 drops per second. Once the entire sample has passed through the cartridge, rinsed the cartridge with 2×6 mL of Milli-Q water, and dried using vacuum pump for 30 min. After that, the PFOA was eluted with 6 mL of 0.1% NH_4OH/methanol. Finally, the extracts were evaporated to approximately 300 μL with nitrogen stream at 40 °C before passing them through the 0.2 μm syringe filter and transferred to autosampler vials, then further concentration to dryness. The autosampler vials were reconstituted with 200 μL methanol for further analysis by LC-TOF-MS. All the samples should store in 4 °C until used.

1.2 LC-TOF-MS setting

A Sciex Exion HPLC coupled to a Sciex 5600+ triple TOF MS was used to detect the unspiked water samples. Firstly, 10 μL of extract was injected into the LC fitted with a Raptor C18 column (1.8 μm particle size, 50 mm length, 2.1 mm internal diameter, Restek). Details of the LC conditions employed are provided as Table S2. The TOF-MS is equipped with a Turbo V source operated in negative mode using electrospray ionization (ESI) at a voltage of −4500 V. The curtain gas and nebulizer gas (source gas 1) were both 25 psi, while the drying gas (source gas 2) was 35 psi. The CAD gas was set to medium, and the temperature was 450 °C. Mass spectrometric data was acquired using automatic information-dependent acquisition (IDA) with two experiment types: (i) survey scan, which provided TOF-MS data, and (ii) dependent product ion scan using a collision energy of -40 V and a collision spread of 30 V. PFOA was quantified in Multiquant 2.0 using MS/MS transitions and retention time for identification (Table S3).

2. Tables and Figures

2.1 Surface characterisation

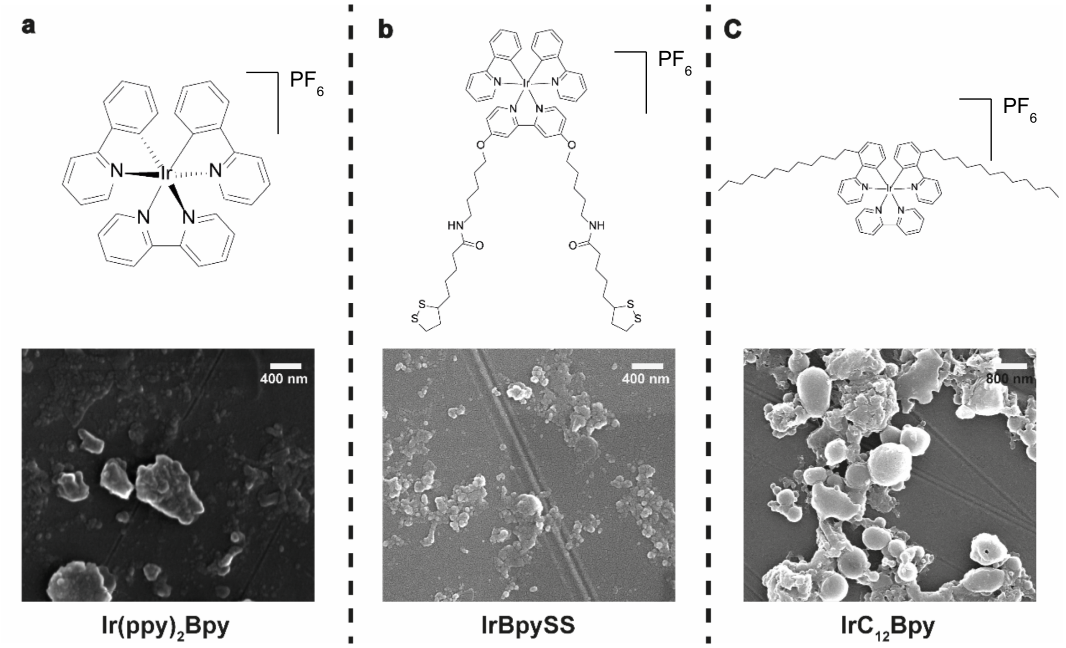

Figure S1. Chemical structure and SEM images of (a) Ir(ppy)2bpy@Au, (b) IrbpySS@Au and (c) IrC12bpy@Au deposited as acetonitrile solutions on Au surfaces.

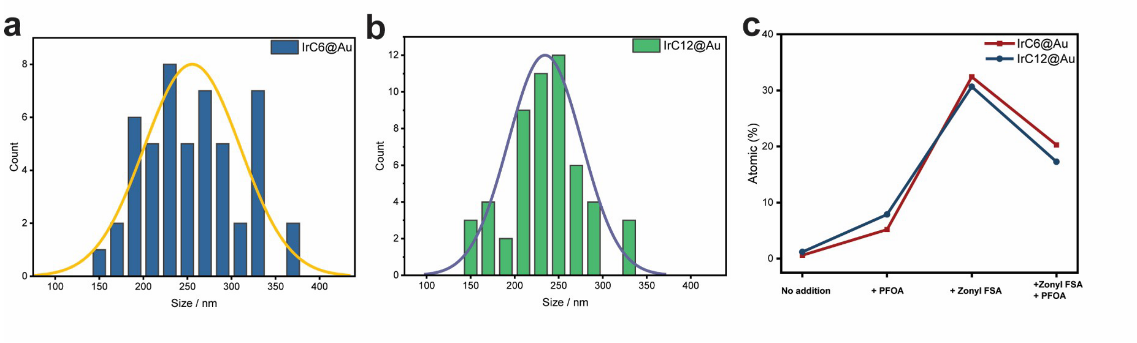

Figure S2. Size distributions of (a) IrC6@Au and (b) and IrC12@Au from SEM images (Figure 2) with N>50. (c) Variation of fluorine composition in different substrates.

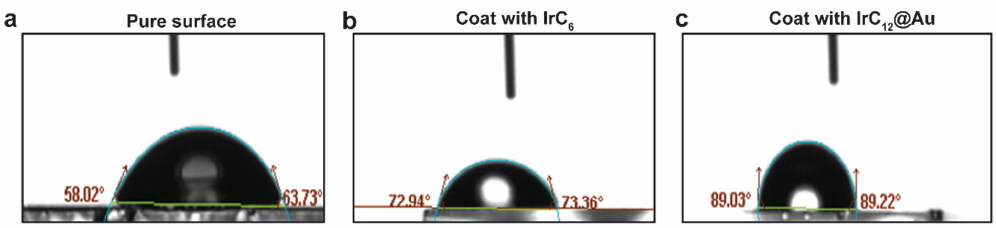

Figure S3. Representative images of the contact angle observed between the gold surface and water droplet a) uncoated gold surface, b) IrC_6@Au and c) IrC_{12}@Au.

Table S1. DLS size analysis of acetonitrile solutions of IrC_6 and IrC_{12} PDI: polydispersity index.

	PDI	Z-ave (d.nm)	Size by Intensity (nm)	Size by Number (nm)
IrC_6	0.12	210±6	150±39	200±60
IrC_{12}	0.33	190±3	140±30	160±33

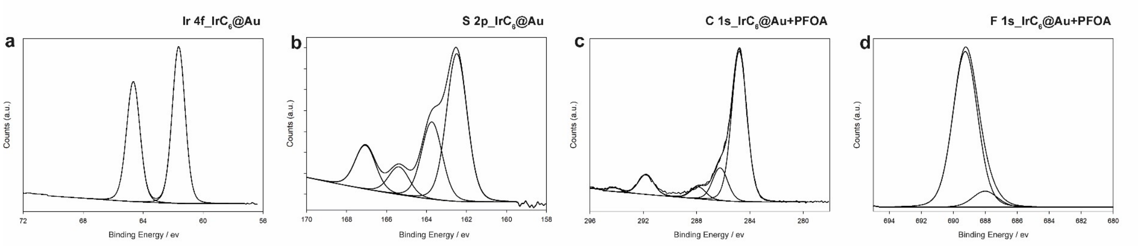

Figure S4. XPS spectrum of (a) Ir 4f spectrum of IrC6@Au, (b) S 2p spectrum of IrC6@Au, (c) C 1s spectrum of IrC6@Au with PFOA (PFOA concentration: 100 mg/L) and (d) F 1s spectrum of IrC6@Au with PFOA (PFOA concentration: 100 mg/L).

(a)

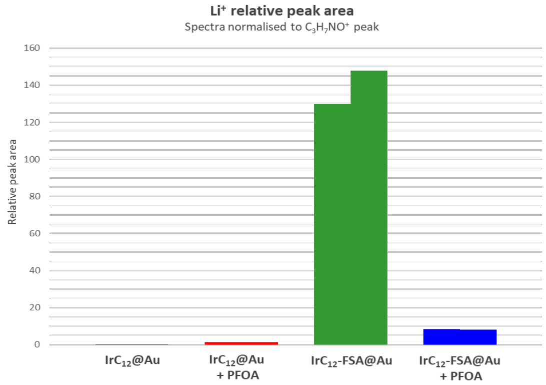

(b)

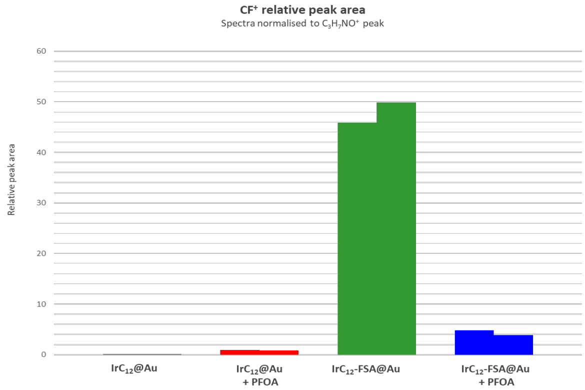

Figure S5: (a) Relative CF^+ peak areas, common to both PFOA and Zonyl-FSA, for different substrates. (b) Relative peak areas of the Li^+ peak unique to Zonyl-FSA

2.2 LC-MS analysis

Table S2. Liquid chromatography elution program

<table>
<tr><td>Mobile Phase</td><td colspan="3">A: 5 mM ammonium formate in water
B: 5 mM ammonium formate in methanol</td></tr>
<tr><td>Column</td><td colspan="3">Restek Raptor C18 column 1.8 µm particle size, 50 mm length, 2.1 mm internal diameter</td></tr>
<tr><td>Flow Rate</td><td colspan="3">0.4 mL/min</td></tr>
<tr><td rowspan="6">Gradient</td><td>Time</td><td>A (%)</td><td>B (%)</td></tr>
<tr><td>0.00</td><td>80</td><td>20</td></tr>
<tr><td>6</td><td>5</td><td>95</td></tr>
<tr><td>6.5</td><td>5</td><td>95</td></tr>
<tr><td>6.51</td><td>80</td><td>20</td></tr>
<tr><td>8</td><td>80</td><td>20</td></tr>
</table>

Table S3 MS/MS transitions and PFOA retention times

**Compound**	**MS/MS Transition**	**Retention Time**
**Native Compounds**		
PFOA	413.16 → 369.12	4.51
**Internal Standards**		
M8-PFOA	421.3 → 377.12	4.50
**Recovery determination standard**		
M4-PFOA	417 → 372	4.50

2.3 Photophysical analysis

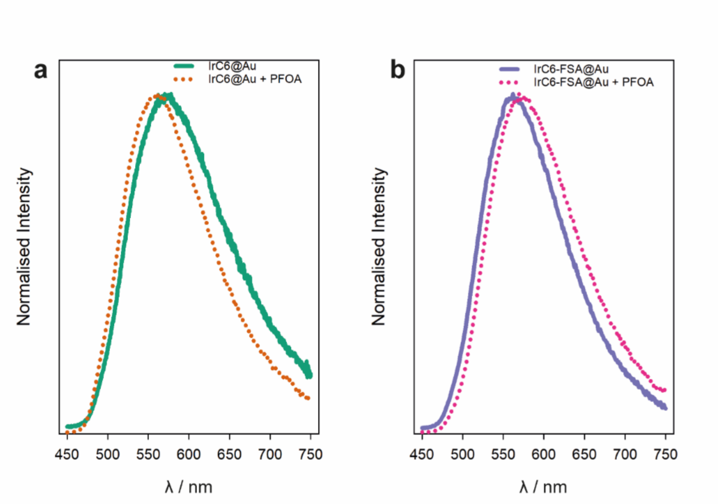

Figure S6. Normalised emission spectra of (a) IrC_6@Au and (b) IrC_6-FSA@Au upon addition of PFOA,: 1g/L for IrC_6@Au and 10 mg/L for IrC_6-FSA@Au; λ_{exc} = 375 nm.

Table S4. Photophysical properties of Ir (III) complexes in acetonitrilesolution upon mixing without or with Zonyl FSA and with the addition of PFOA. PFOA solution added: 41.4 mg/L. Estimated errors λ_{em} ± 1 nm, and τ ± 10%.

Name	λ_{em} (nm)	τ (ns)		χ^2
IrC_6	608	30 (21%)	55 (79%)	1.11
IrC_6 + PFOA	608	47 (77%)	81 (23%)	1.18
IrC_6 + FSA	612	33 (26%)	69 (74%)	1.12
IrC_6 + FSA + PFOA	610	38 (45%)	63 (55%)	1.18
IrC_{12}	611	35 (39%)	62 (61%)	1.13
IrC_{12} + PFOA	608	49 (85%)	83 (15%)	1.20
IrC_{12} + FSA	615	33 (24%)	74 (76%)	1.14
IrC_{12} + FSA + PFOA	611	39 (36%)	61 (64%)	1.17

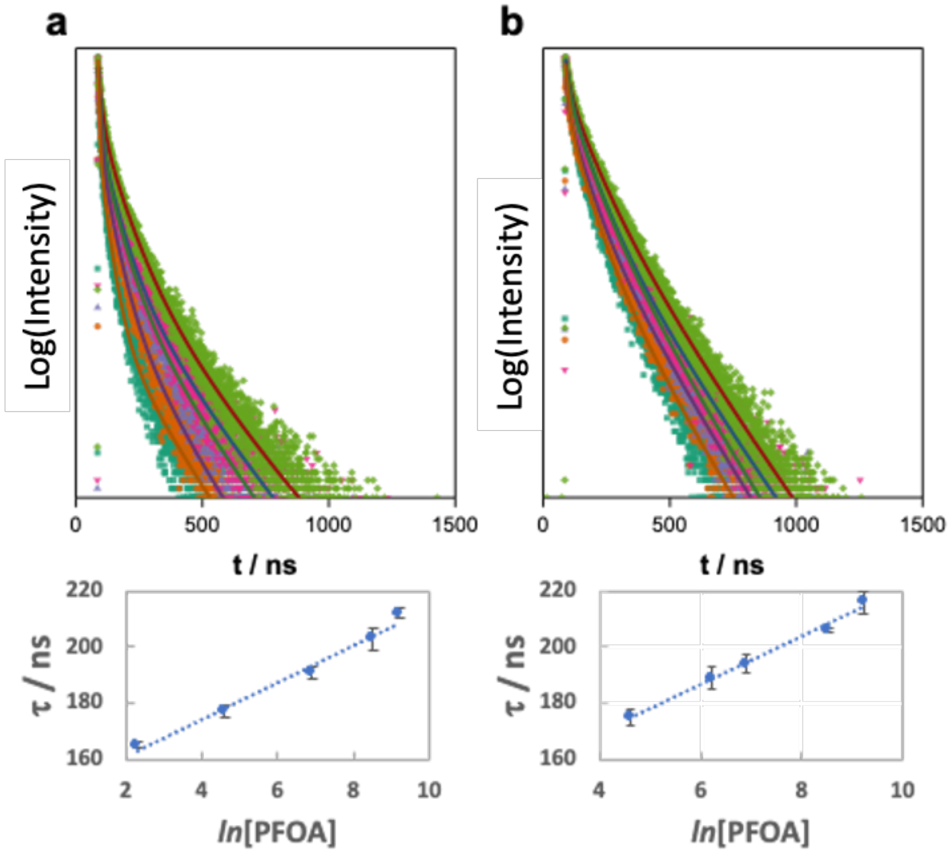

Figure S7. Luminescence lifetime decays (normalised plots -showing fitting in solid line) of (a) IrC6@Au and (b) IrC12@Au surfaces upon addition of PFOA, $\chi2 = 1.0 \pm 0.2$, accompanied by plots of the luminescence lifetime (major component) with ln[PFOA]. The minor lifetime component showed same dependency.

Table S5 Luminescence lifetime of IrC6-FSA@Au and IrC12-FSA@Au upon addition of PFOA $\chi 2$ within 1.0 ± 0.2, n=3

C_{PFOA} (mg/L)	IrC6-FSA@Au τ /ns		C_{PFOA} (mg/L)	IrC12-FSA@Au τ /ns	
1000	56±3 (15%)	187±12 (85%)	1000	65±8 (19%)	196±14 (81)
100	68±7 (22%)	237±13 (78%)	100	83±12 (20%)	249±21 (80%)
10	76±1 (25%)	307±7 (75%)	10	93±7 (25%)	308±3 (75%)
1	86±3 (24%)	348±4 (76%)	1	106±10 (20%)	366±12 (80%)
0.1	102±5 (19%)	409±16 (81%)	0.1	129±2 (18%)	434±11 (82%)
0	119±6 (19%)	446±5 (81%)	0.01	164±3 (22%)	469±3 (78%)
			0	167±5 (20%)	502±4 (80%)

Table S6. PFOA concentration in different countries' drinking water.

Country	Sampling site	C_{PFOA} (ng/L)	Ref.
Ireland	Tap water samples were collected from home and office connected to municipal water supplies	0.04-1.76	3
Norway	3 drinking water samples were collected from the tap in households receiving water from different water works	0.65-2.5	4
German	5 tap water samples were collected from potable water treatment plant located at the federal state of Hesse	0.16-1.9	5
Belgium	Drinking water samples were collected from local origin	1-5	6
Netherlands	Drinking water samples were collected from the cities in the vicinity from the PFAS production plant. The cities include Zwijndrecht, Dordrecht, Papendrecht, Sliedrecht, Utrecht, Wageningen.	<0.3-2.7	7
Italy	Samples were collected from North of Milan (industrialized area)	10-47	8

Spain	30 drinking water samples collected in 10 Catalonia area drinking water treatment plants	<0.40-9.6	9
US	Samples collected from 25 drinking water treatment plants located in 24 contiguous states	0.56-104	10
China	The tap water samples were collected from 79 cities in 31 provincial-level administrative regions throughout China	n.d. - 26.33	11

3. Syntheses of Ir (III) Complexes

BpySS

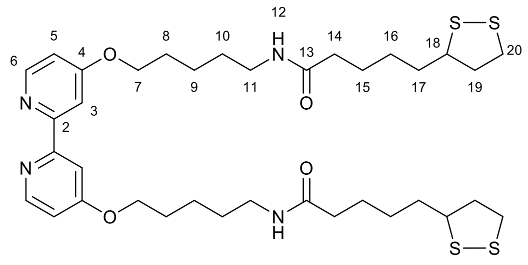

Scheme S1: Structure of BpySS

1-Hydroxybenzotriazole hydrate (0.50 g, 3.7 mmol, 1.7 eq) and α-lipoic acid (0.46 g, 2.2 mmol, 1 eq), in dry DMF (10 mL) was cooled to 0-5 ^{o}C, upon which 1-ethyl-3-(3-dimethylaminopropyl)carbodiimide (EDC) (0.57 g, 3.7 mmol, 1.7 eq) was added dropwise and allowed to stir until full dissolution of EDC. The solution was warmed to room temperature, and a solution of N-ethylmorpholine (0.26 g, 2.3 mmol, 1 eq) and 4,4'-di-(5-amino-1-pentoxy)-2,2'-bipyridine (0.36 g, 1.00 mmol, 0.45 eq) in dry DMF (10 mL) was added dropwise and the reaction stirred overnight. The resulting cream precipitate was filtered, washed with Diethyl-ether and dried in air to yield BpySS as a white powder (0.36 g, 0.48 mmol, 48%) ^{1}H-NMR (400 MHz, CDCl3): δ = 8.39 (d, J =5.6 Hz, 2H, H-6), 7.87 (d, J =2.5 Hz, 2H, H-3), 6.75 (dd, J =5.7, 2.5 Hz, 2H, H-5), 5.41 (s, 2H, NH), 4.07 (t, J =6.3 Hz, 4H, H-7), 3.50 (dt, J =13.6, 6.5 Hz, 2H, H-17), 3.22 (q, J =6.5 Hz, 4H, H-11), 3.17 – 2.96 (m, 4H, H-19), 2.38 (dtd, J =13.0, 6.5, 5.4 Hz, 2H, H-18), 2.11 (t, J =7.4 Hz, 4H, H-13), 1.89-1.75(m, 6H, H-18', H-10) 1.63-1.57 (m,

8H, H-14, H-16), 1.51 – 1.30 (m, 12H, H-8, H-9, H-15). HRMS (ESI): m/z calculated for $[C_{36}H_{54}N_4O_4S_4 + H]^+$ theoretical mass 735. 3100 measured mass 735. 3135

IrC_6

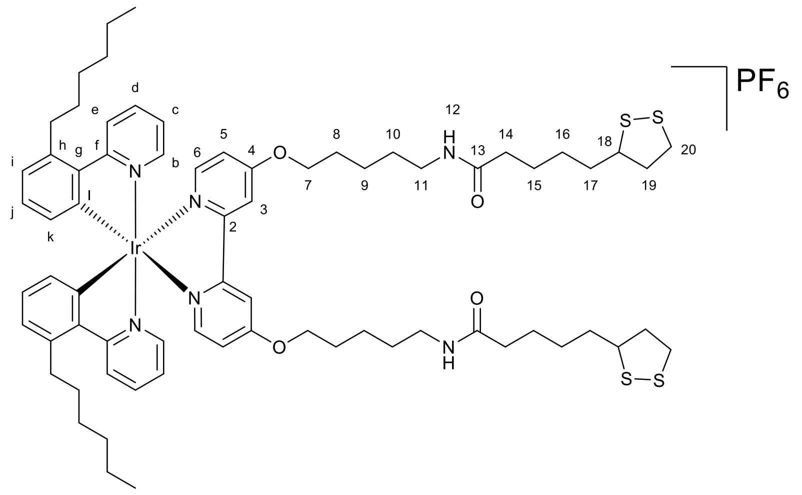

Scheme S2: Structure of IrC_6

2-(2-n-hexylphenyl)pyridine

The method was based on an adaptation of the method by Ackermann *et* al.[1] A suspension of $[RuCl_2(p\text{-cymene})]_2$ (157 mg, 0.256 mmol, 0.0256 eq), 1-$AdCO_2H$ (547.2 mg, 3.04 mmol, 0.0304 eq), K_2CO_3 (2.80 g, 20 mmol, 2 eq), 2-phenylpyridine (1.55 g, 10 mmol, 1 eq) and 1-bromohexane (4.95 g, 30 mmol, 3 eq) were dissolved in N-methyl-2-pyrrolidone (40 ml). The mixture was then stirred at 100 °C under N_2 for 24 h. The aqueous phase was then extracted with EtOAc (2 x 50 ml) and the combined organic layers were washed with H_2O (50 ml) and brine (2 x 50 ml) and dried over $MgSO_4$, filtered and concentrated *in vacuo*. The product was then purified using silica column chromatography (hexane:EtOAc 5:1) and gave 2-(2-n-hexylphenyl)pyridine as a yellow-brown oil (450 mg, 60.5 %). ^{1}H-NMR (400 MHz, $CDCl_3$): δ = 8.68 (ddd, 4.9, 1.8, 1.0 Hz, 1H, *H-b*), 7.73 (td, 7.7, 1.8 Hz, 1H, *H-d*), 7.41-7.18 (m, 6H, *H-c,e,l,k,j.i*), 2.73-2.66 (m, 2H, *CH_2*), 1.48-1.38 (m, 2H, *CH_2*), 1.24-1.07 (m, 6H, *CH_2*), 0.82 (t, J = 6.78 Hz, *CH_3*). ^{13}C-NMR (100 MHz, $CDCl_3$): δ = 160.4 (C-f), 149.1 (C-b), 140.8 (C-d), 140.4 (C-e), 136.1 (C-g), 129.7 (C-I,j), 128.3 (C-k), 125.7 (C-c), 124.1 (C-l), 121.6 (CH_2), 32.9 (CH_2),

31.5 (CH_2), 31.2 (CH_2), 29.1 (CH_2), 22.5 (CH_2), 14.1 (CH_3) HRMS (ESI): m/z calculated for $[C_{17}H_{21}N^+]H^+$ = 240.1757 found 240.1748

The synthesis of the iridium dimers is based on the method set out by Watts *et al.*[2] Iridium trichloride hydrate (0.0.155 g, 0.519 mmol, 1 eq) was added to 2-(2-n-hexylphenyl)pyridine (0.300 g, 1.25 mmol, 2.4 eq) and dissolved with 2-ethoxyethanol (20 ml) and water (10 ml). The solution was then left to reflux for 24 h. Once the solution had cooled to room temperature it was filtered and the yellow precipitate was collected on a glass filter frit. The precipitate was then washed with 95% ethanol and methanol to give yellow crystals of $[Ir(ppyhex)_2Cl]_2$ (0.801 g, 61.2%, 0.765 mmol). $[Ir(ppyhex)_2Cl]_2$ (40 mg, 0.028 mmol, 1 eq) and bpySS (53 mg, 0.057 mmol, 2 eq) were suspended in MeOH/DCM (20 ml, 3/1, v/v) and heated at reflux for 4 hours. The mixture was then cooled, filtered and the solvent removed *in vacuo*. Ammonium hexafluorophosphate (48.9 mg, 3.00 mmol) was added in methanol (2 ml) and stirred for 1 h. The solution was then cooled on ice filtered and washed in H_2O. The solid was then triturated in hexane and filtered to give IrC_6 (0.030 g, 64%, 0.021 mmol). ^{1}H-NMR (400 MHz, acetone-d_6): 8.82 (d, J = 2.5 Hz, 2H, H-3), 8.24 (dd, J = 8.2, 0.9 Hz, 2H, H-d), 8.00 – 7.94 (m, 4H, H-c,e), 7.65 (d, J = 6.4 Hz, 2H, H-6), 7.16 (ddd, J = 6.0, 2.5 Hz, 2H, H-b), 7.10 (dd, J = 6.4, 2.5 Hz, 2H, H-5), 6.85 (dd, J = 7.5, 1.3 Hz, 2H, H-i), 6.73 (dd, J = 7.5 Hz, 2H, H-j), 6.12 (dd, J = 7.6, 1.3 Hz, 2H, H-k), 4.48 (m, 4H, H-7), 3.55 (td, J = 8.7, 1.8 Hz, 2H, H-18), 3.27 – 3.04 (m, 12H, H-10,11,20), 2.44 (dp, J = 12.5, 6.4 Hz, 2H, H-19’), 2.21 (t, J = 7.3 Hz, 4H, H-14), 1.91-1.80 (m,2H, H-19, CH_2), 1.76 – 1.68 (m, 8H, H-8, CH_2), 1.64-1.52 (m, 16H, H-9,14,15,16), 1.43 – 1.24 (m, 12H, CH_2), 0.89 (t, J = 7.2, 6.8 Hz, 6H, H-i). ^{13}C-NMR (100 MHz, acetone-d6): δ = 173.1 (C-12), 168.7 (C-4), 168.5 (C-f), 159.1 (C-2), 155.3 (C-l), 151.2 (C-6), 150.7 (C-e), 142.7 (C-g), 142.6 (C-h), 138.9 (C-c), 130.6 (C-k), 130.2 (C-j), 126.9 (C-i), 124.4 (C-b), 123.9 (C-d), 116.4 (C-5), 113.1 (C-3), 71.7 (C-7), 57.5 (C-17), 41.1 (C-18), 39.2 (C-11), 39.1 (C-19), 36.5 (C-10), 35.4 (C-16), 32.5 (CH_2), 31.2 (C-8), 30.4 (CH_2), 30.2 (CH_2), 28.8 (CH_2), 26.5 (C-15), 23.7 (C-9), 23.5 (CH_2), 14.4 (CH_3). HRMS (ESI): m/z calculated for monomer $[C_{70}H_{94}IrN_6O_4S_4]^+$ theoretical mass 1403.5921 calculated mass 1403.5825. λ_{max} / nm (ε / 10^4 M^-

[1] cm^{-1}): 227 (3.1), 258 (2.7), 297 (1.2), 347 (0.5). Found: C, 55.6; H, 6.5; N, 5.45 Calc. $C_{70}H_{94}IrN_6O_4S_4PF_6.C_6H_{14}$: C, 55.8, H, 6.7 N 5.1 %

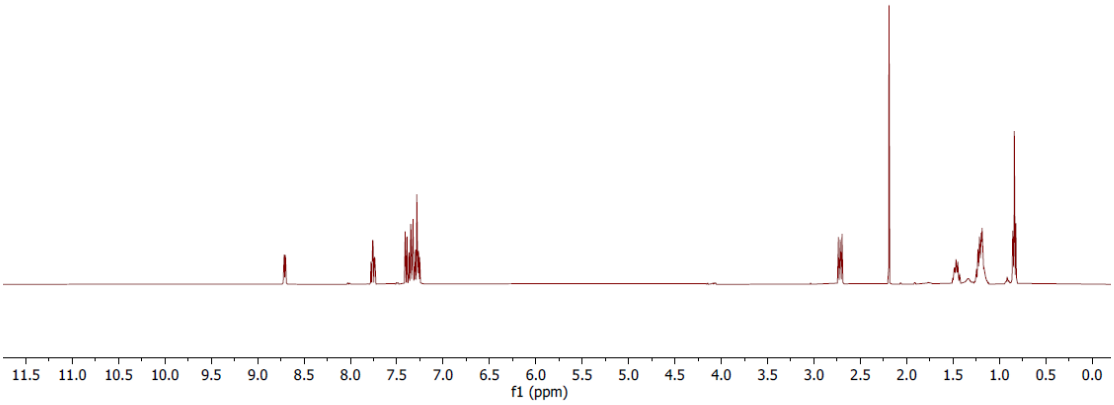

Figure S8: 400 MHz ^{1}H NMR of 2-(2-n-hexylphenyl)pyridine in $CDCl_3$.

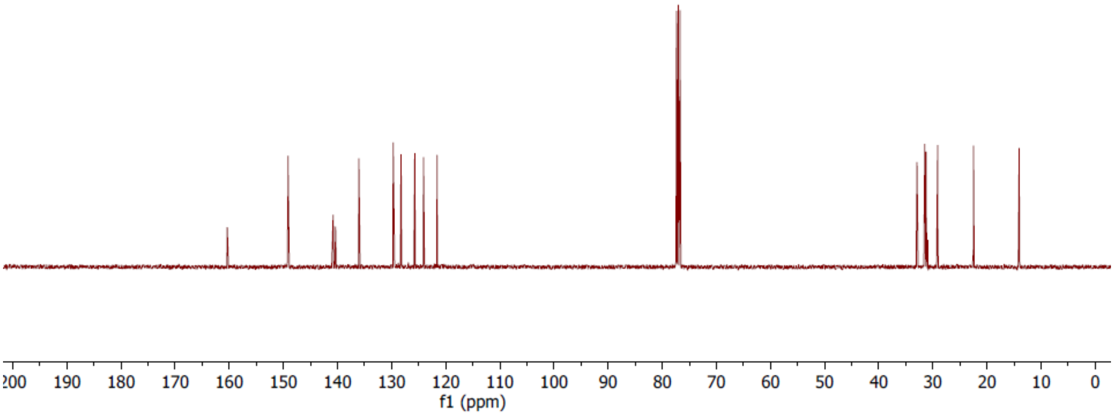

Figure S9: 101 MHz ^{13}C NMR of 2-(2-n-hexylphenyl)pyridine in $CDCl_3$.

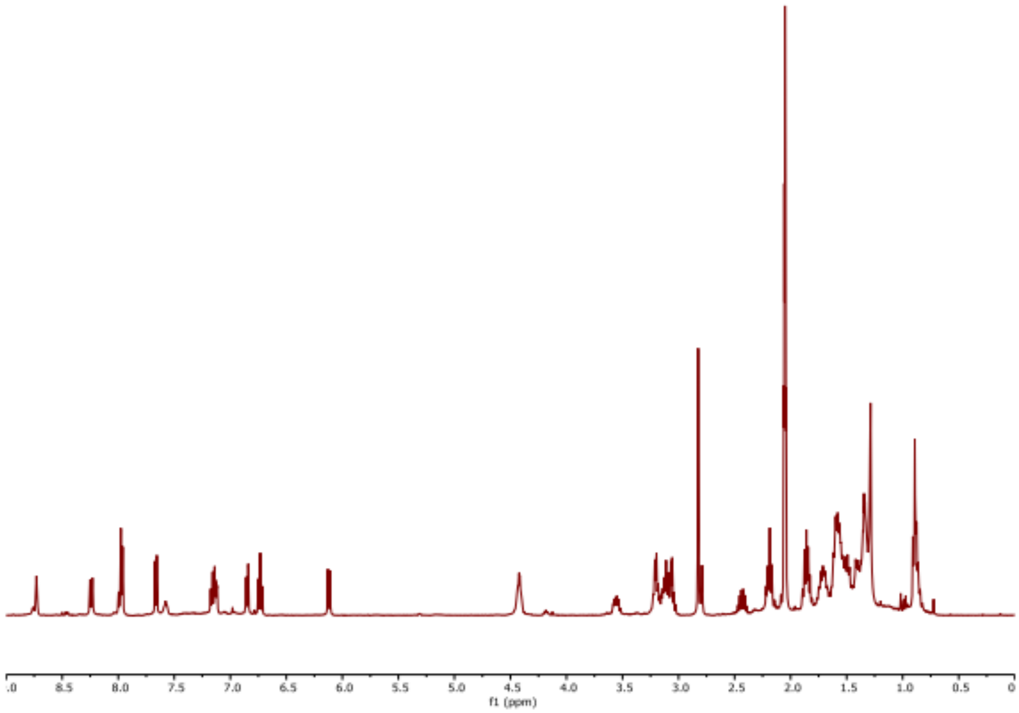

Figure S10: 400 MHz ^{1}H NMR of IrC_6 in acetone-d_6.

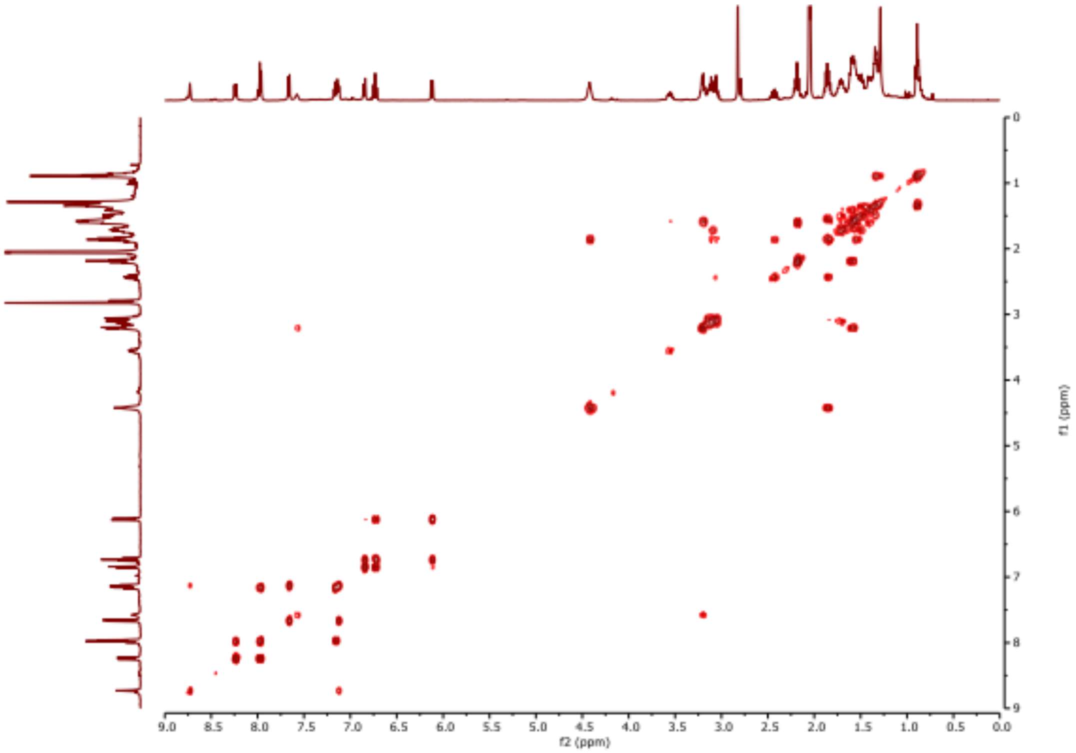

Figure S11: COSY of IrC_6 in acetone-d_6.

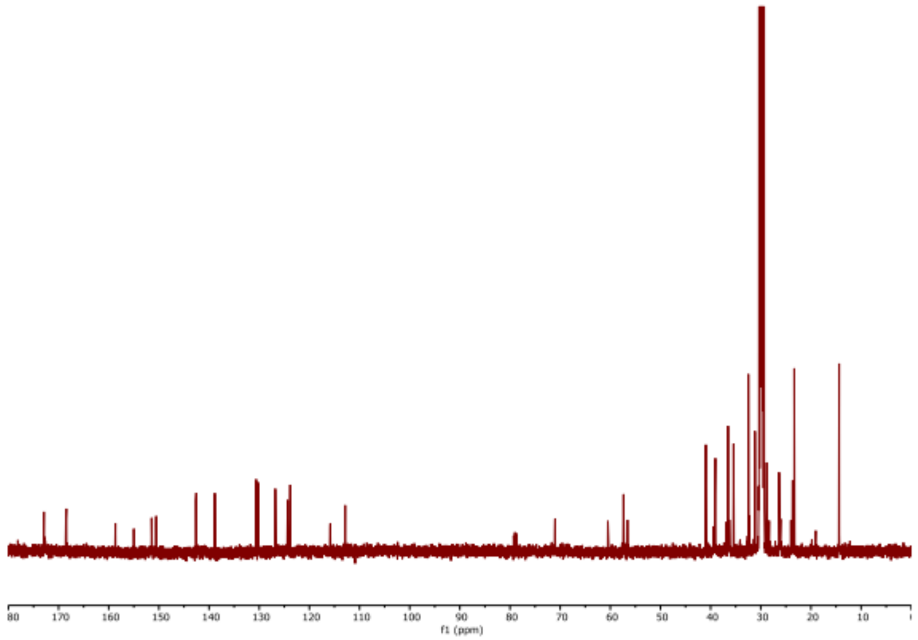

Figure S12: 101 MHz ^{13}C NMR of IrC_6 in acetone-d_6.

IrC_{12}

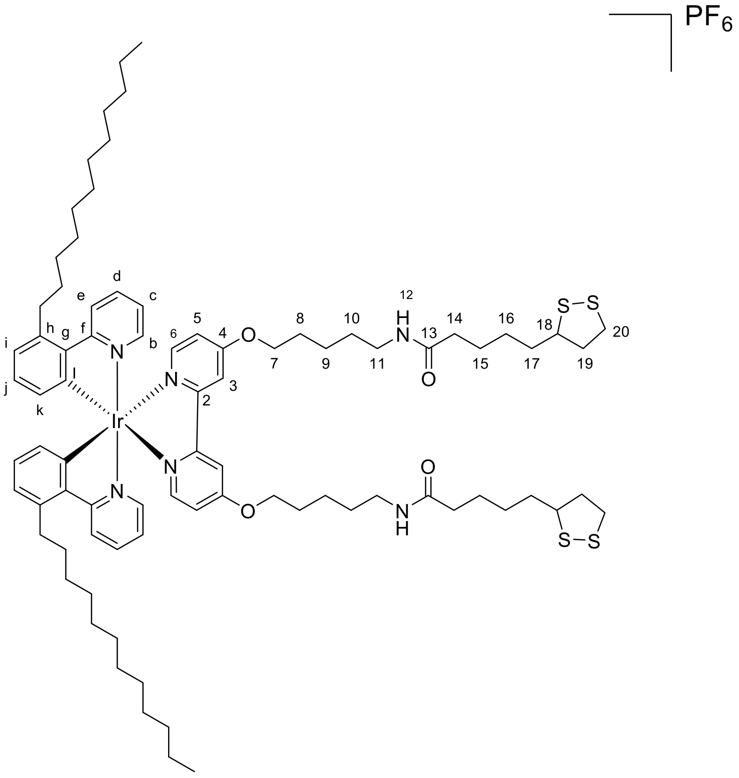

Scheme S3: Structure of IrC_{12}

2-(2-n-dodecylphenyl)pyridine

The method was based on an adaptation of the method by Ackermann *et* al.[1] A suspension of $[RuCl_2(p\text{-cymene})]_2$ (157 mg, 0.256 mmol, 0.0256 eq), 1-AdCO_2H (547.2 mg, 3.04 mmol, 0.0304 eq), K_2CO_3 (2.80 g, 20 mmol, 2 eq), 2-phenylpyridine (1.55 g, 10 mmol, 1 eq) and 1-bromododecane (4.95 g, 20 mmol, 2 eq) were dissolved in N-methyl-2-pyrrolidone (40 ml). The mixture was then stirred at 100 °C under N_2 for 24 h. The aqueous phase was then extracted with EtOAc (2 x 50 ml) and the combined organic layers were washed with H_2O (50 ml) and brine (2 x 50 ml) and dried over $MgSO_4$, filtered and concentrated *in vacuo*. The product was then purified using silica column chromatography (hexane:EtOAc 5:1) and gave 2-(2-n-dodecylphenyl)pyridine as a yellow-brown oil (455 mg, 60.5 %).^{1}H-NMR (400 MHz, $CDCl_3$): δ = 8.67 (ddd, J = 4.8, 1.8, 0.9 Hz, 1H, *H-b*), 7.72 (td, J = 7.7, 1.8 Hz, 1H, *H-d)*, 7.37 – 7.21 (m, 6H, *H-c,e,l,k,j,i*), 2.71 – 2.63 (m, 2H, *CH_2*), 1.46 – 1.36 (m, 2H, *CH_2*), 1.24 (s, 40H, *CH_2*), 0.86 (t, J = 6.7 Hz, 3H, *CH_3*). ^{13}C-NMR (100 MHz, $CDCl_3$): 160.5 (C-f), 149.3 (C-b), 141.0 (C-d), 140.5 (C-e), 136.2 (C-g), 129.9 (C-j), 129.9 (C-k), 128.4 (C-i), 125.9 (C-c), 124.3 (C-i), 121.7 (C-l), 33.1 (CH_2), 32.1 (CH_2), 31.4 (CH_2), 29.9 (CH_2), 29.8 (CH_2), 29.8 (CH_2), 29.6 (CH_2), 29.6 (CH_2), 29.5 (CH_2), 29.4 (CH_2), 22.9 (CH_2), 14.3 (CH_3). HRMS (ESI): m/z Theoretical mass for $[C_{35}H_{57}N^+]H^+$ = 492.4564 Calculated mass = 492.4583

The synthesis of the iridium dimers is based on the method set out by Watts *et al.*[2] Iridium trichloride hydrate (0.0.155 g, 0.519 mmol, 1 eq) was added to 2-(2-n-dodecylphenyl)pyridine (0.300 g, 1.25 mmol, 2.4 eq) and dissolved with 2-ethoxyethanol (20 ml) and water (10 ml). The solution was then left to reflux for 24 h. Once the solution had cooled to room temperature it was filtered and the yellow precipitate was collected on a glass filter frit. The precipitate was then washed with 95% ethanol and methanol to give yellow crystals of $[Ir(ppy)_2Cl]_2$ (0.801 g, 61.2%, 0.765 mmol). $[Ir(ppydodec)_2Cl]_2$ (40 mg, 0.023 mmol, 1 eq) and bpySS (53 mg, 0.057 mmol, 2.5 eq) were suspended in MeOH/DCM (20 ml, 3/1, v/v) and heated at reflux for 4 hours. The mixture was then cooled, filtered and the solvent removed *in vacuo*. Ammonium hexafluorophosphate (48.9 mg, 3.00 mmol) was added in methanol (2 ml) and stirred for 1 h. The solution was then cooled on ice filtered and washed in H_2O. The solid was then triturated

in hexane and filtered to give IrC_{12} (0.039 g, 55%, 0.025 mmol). ^{1}H-NMR (400 MHz, acetone-d_6): 9.04 (d, J = 2.5 Hz, 2H, H-3), 8.22 (dd, J = 8.2, 0.9 Hz, 2H, H-d), 8.01 – 7.93 (m, 4H, H-c,e), 7.63 (d, J = 6.0, 2.5 Hz, 2H, H-b), 7.16 (ddd, J = 7.4, 1.0 Hz, 2H, H-b), 7.06 (dd, J = 6.4, 2.5 Hz, 2H, H-5), 6.82 (dd, J = 7.5, 1.3 Hz, 2H, H-i), 6.71 (dd, J = 7.5 Hz, 2H, j), 6.12 (dd, J = 7.5, 1.3 Hz, 2H, H-k), 4.54 (q, J = 8.9, 8.4 Hz, 4H, H-7), 3.59 – 3.48 (m, 2H, H-17), 3.22 – 2.97 (m, 12H, H-10,11,19), 2.41 (dq, J = 12.4, 6.3 Hz, 2H, H-18'), 2.23 (t, J = 7.3 Hz, 4H, H-13), 1.89 – 1.76 (m, 6H, H-18, CH_2), 1.75 – 1.63 (m, 8H, H-8, CH_2), 1.63 – 1.42 (m, 16H, H-9,14,15,16), 1.42 – 1.20 (m, 36H, CH_2), 0.87 (t, J = 7.0, 6H). ^{13}C-NMR (100 MHz, acetone-d_6): δ = 173.1 (C-12), 168.6 (C-4), 168.5 (C-f), 158.9 (C-2), 155.2 (C-l), 151.1 (C-6), 150.5 (C-e), 142.6 (C-g), 142.6 (C-h), 138.8 (C-c), 130.6 (C-k), 130.2 (C-j), 126.8 (C-i), 124.3 (C-b), 123.9 (C-d), 116.4 (C-5), 113.0 (C-3), 71.5 (C-7), 57.4 (C-10), 41.0 (C-18), 39.2 (C-11), 39.0 (C-19), 36.5 (C-13), 36.5 (CH_2), 36.4 (CH_2), 35.4 (C-16), 32.7 (CH_2), 31.2 (CH_2), 30.4 (CH_2), 30.3 (CH_2), 30.3 (CH_2), 30.1 (CH_2), 29.5 (C-8), 28.8 (CH_2), 26.4 (C-15), 23.6 (C-9), 23.3 (CH_2), 14.4 (CH_3). HRMS (ESI): m/z calculated for monomer $[C_{82}H_{118}IrN_6O_4S_4]^+$ theoretical mass 1571.7727 calculated mass 1571.7953. λ_{max} / nm (ε / 10^4 M^{-1} cm^{-1}): 254 (3.7), 313 (1.4), 348 (0.7). Found: C, 57.6; H, 7.15; N, 4.9. Calc. : C, 57.35; H, 6.9; N, 4.9%

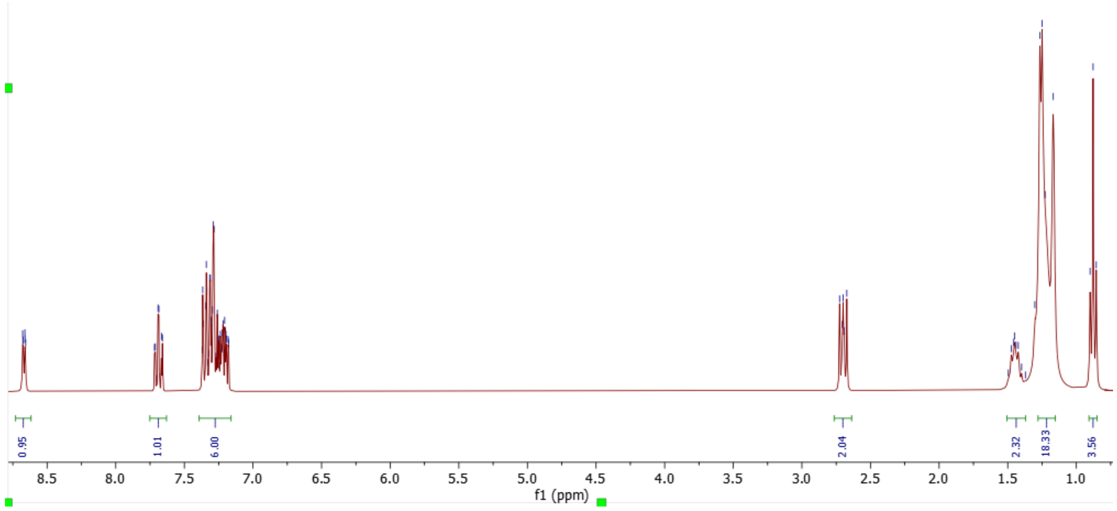

Figure S13: 400 MHz ^{1}H NMR of 2-(2-n-dodecylphenyl)pyridine in $CDCl_3$.

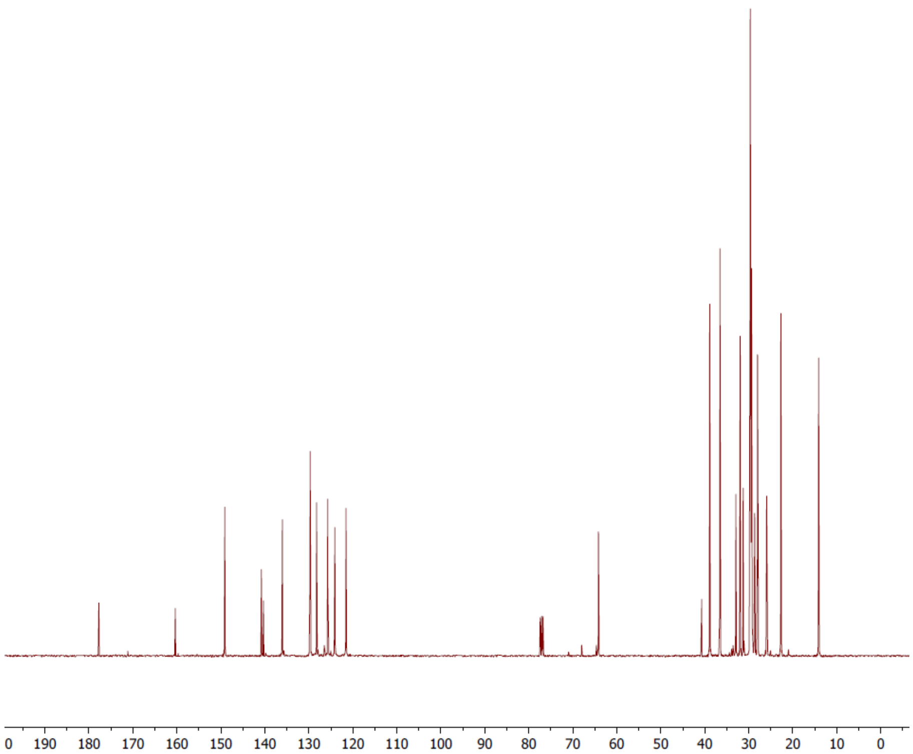

Figure S14: 101 MHz ^{13}C NMR of 2-(2-n-dodecylphenyl)pyridine in $CDCl_3$.

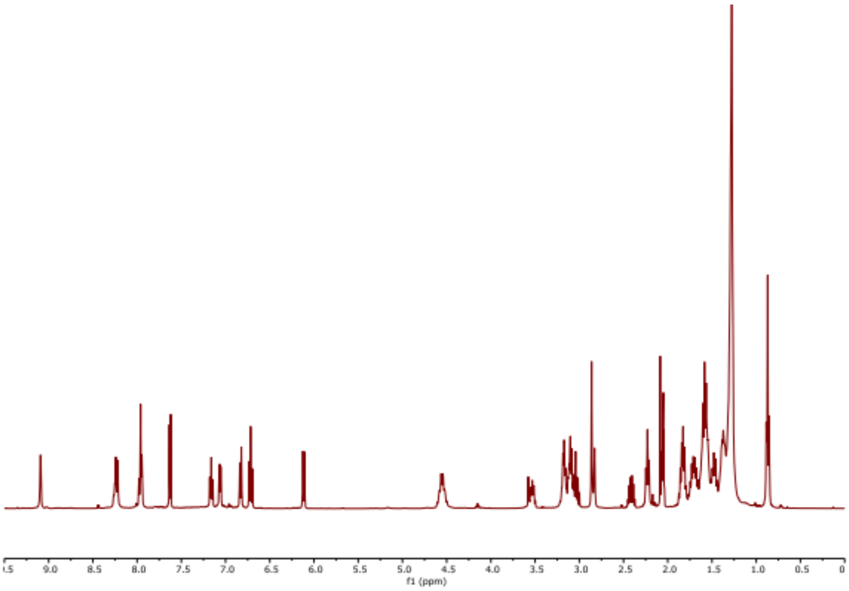

Figure S15: 400 MHz ^{1}H NMR of IrC_{12} in acetone-d_6.

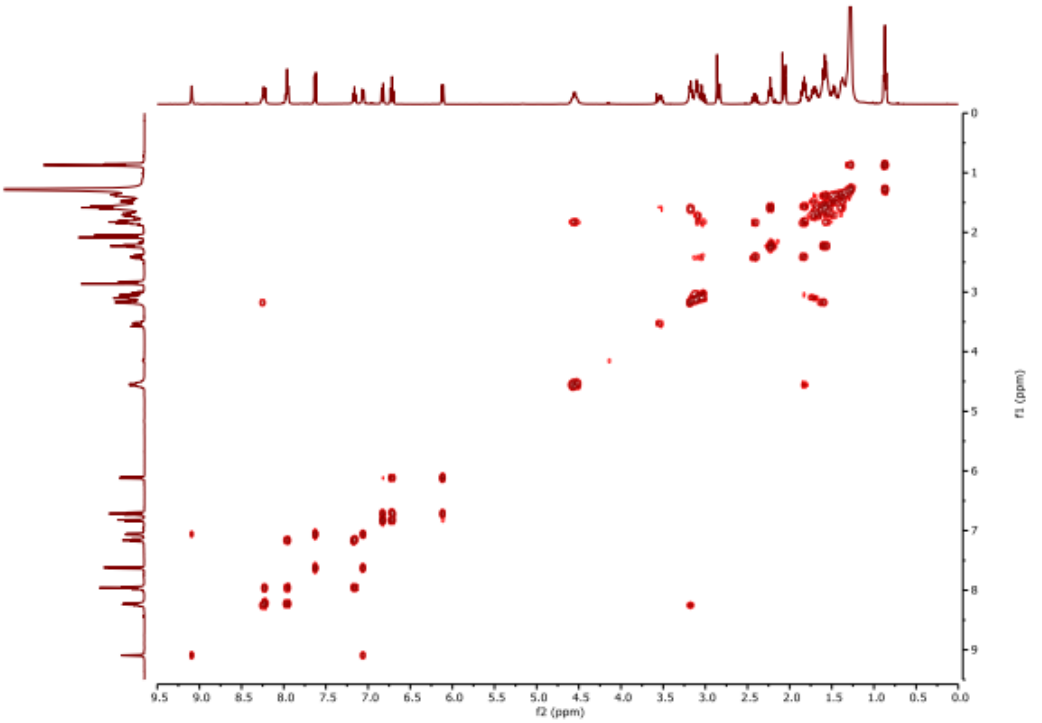

Figure S16: COSY of IrC_{12} in acetone-d_6.

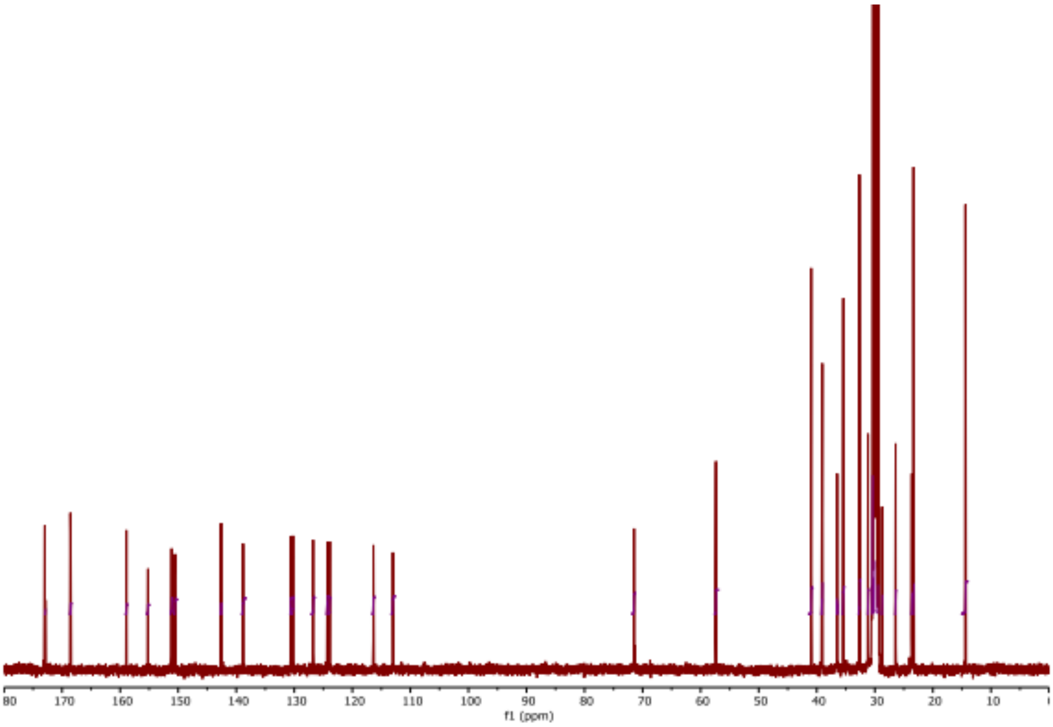

Figure S17: 101 MHz ^{13}C NMR of IrC_{12} in acetone-d_6.

$IrC_{12}bpy$

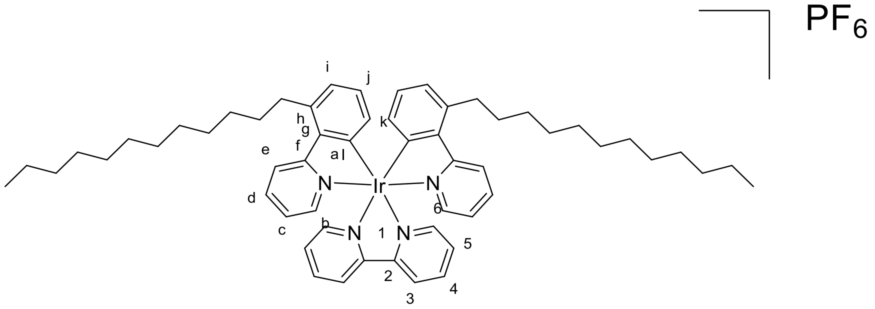

2-(2-n-dodecylphenyl)pyridine (1.08 g, 3.34 mmol, 4.2 eq) was formed as in IrC_{12} an added to Iridium trichloride hydrate (0.235 g, 0.787 mmol, 1 eq) and dissolved with 2-ethoxyethanol (20 ml) and water (10 ml). The solution was then left to reflux for 24 h. Once the solution had cooled to room temperature it was filtered and the yellow precipitate was collected on a glass filter frit. The precipitate was then washed with 95% ethanol and methanol to give yellow crystals of $[Ir(ppydodec)_2Cl]_2$ (0.755 g, 55%, 0.433 mmol). $[Ir(ppydodec)_2Cl]_2$ (25 mg, 0.014 mmol, 1 eq) and bpy (6.7 mg, 0.043 mmol, 3 eq) were suspended in MeOH/DCM (20 ml, 3/1, v/v) and heated at reflux for 4 hours. The mixture was then cooled, filtered and the solvent removed *in vacuo*. Ammonium hexafluorophosphate (48.9 mg, 3.00 mmol) was added in methanol (2 ml) and stirred for 1 h. The solution was then cooled on ice filtered and washed in H_2O. The solid was then triturated in hexane and filtered to give $IrC_{12}bpy$ (20 mg, 71%, 0.020 mmol). ^1H NMR (400 MHz, acetone-d_6) δ 9.00 (dt, J =8.3, 1.0 Hz, 2H, H-3), 8.37 – 8.16 (m, 4H, H-4,b), 8.06 – 7.83 (m, 6H, H-6,c,d), 7.64 (ddd, J =7.7, 5.4, 1.1 Hz, 2H, H-5), 7.12 (ddd, J =7.3, 5.8, 1.3 Hz, 2H, H-e), 6.87 (dd, J =7.5, 1.3 Hz, 2H, H-i), 6.76 (t, J =7.5 Hz, 2H, H-j), 6.13 (dd, J =7.5, 1.3 Hz, 2H, H-k), 3.65 – 3.55 (m, 4H, CH_2), 3.15 – 3.08 (m, 4H, CH_2), 1.81 – 1.65 (m, 6H, CH_2), 1.52 – 1.43 (m, 6H CH_2), 1.27 (s, 24H, CH_2), 0.91 – 0.80 (m, 6H, CH_3). ^{13}C (101 MHz, acetone-d_6) δ 168.3 (C-f), 156.8 (C-2), 154.1 (C-l), 151.0 (C-d), 150.7 (C-6), 142.8 (C-g), 142.5 (C-h), 140.6 (C-4), 139.1 (C-c), 130.5 (C-k), 130.4 (C-j), 129.2 (C-5), 127.1 (C-i), 126.2 (C-3), 124.5 (C-b), 124.0 (C-e), 68.1 (CH_2), 55.0 (CH_2), 49.7 (CH_2), 36.5 (CH_2), 32.6

(CH_2), 31.1 (CH_2), 23.3 (CH_2), 14.4 (CH_3). λ_{max} / nm (ε / 10^4 M^{-1} cm^{-1}): 260 (3.5), 311 (1.9), 348 (0.7). m/z (ESI-TOF) $C_{56}H_{72}N_4Ir$ Calcd: 993.5386 Found: 993.5385

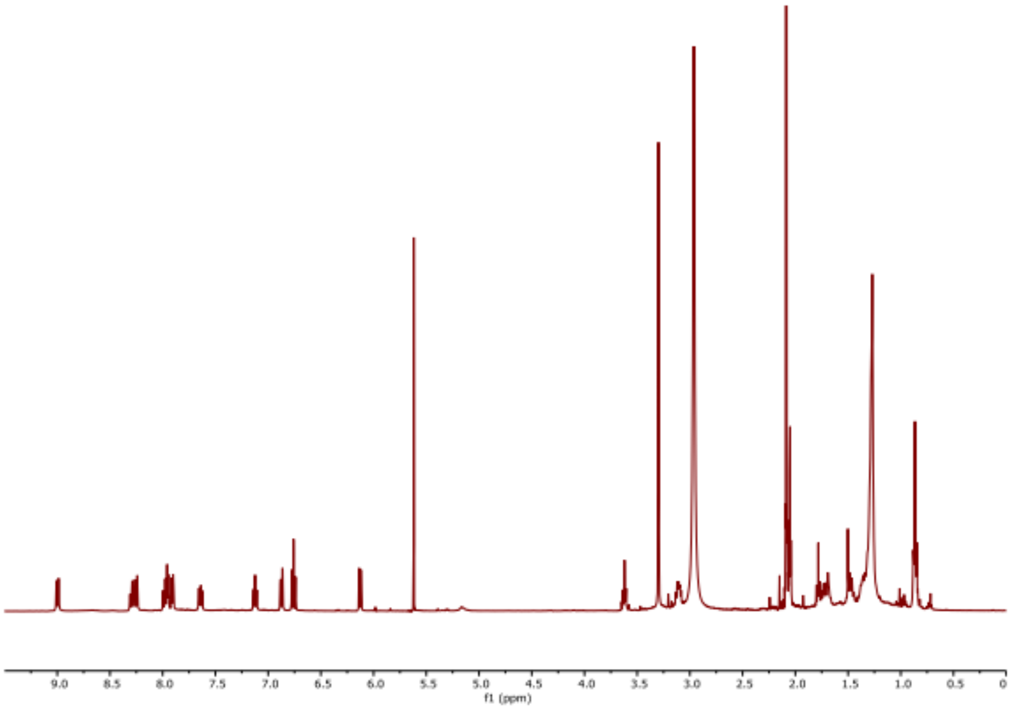

Figure S18: 400 MHz ^{1}H NMR of IrC$_{12}$bpy in acetone-d$_6$.

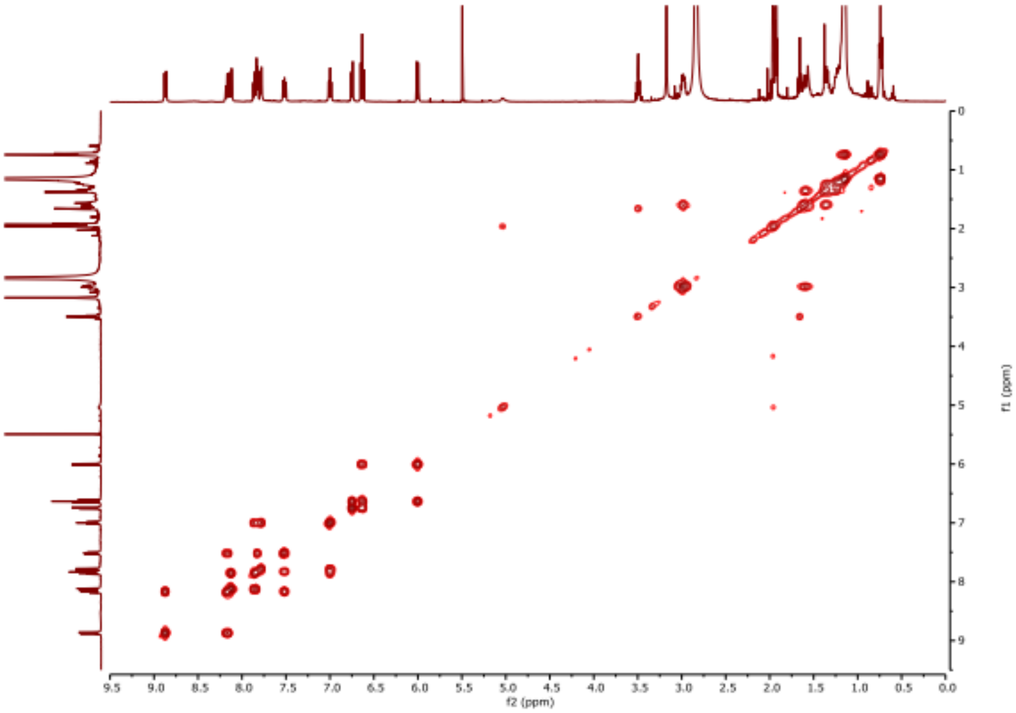

Figure S19: COSY of IrC$_{12}$bpy in acetone-d$_6$.

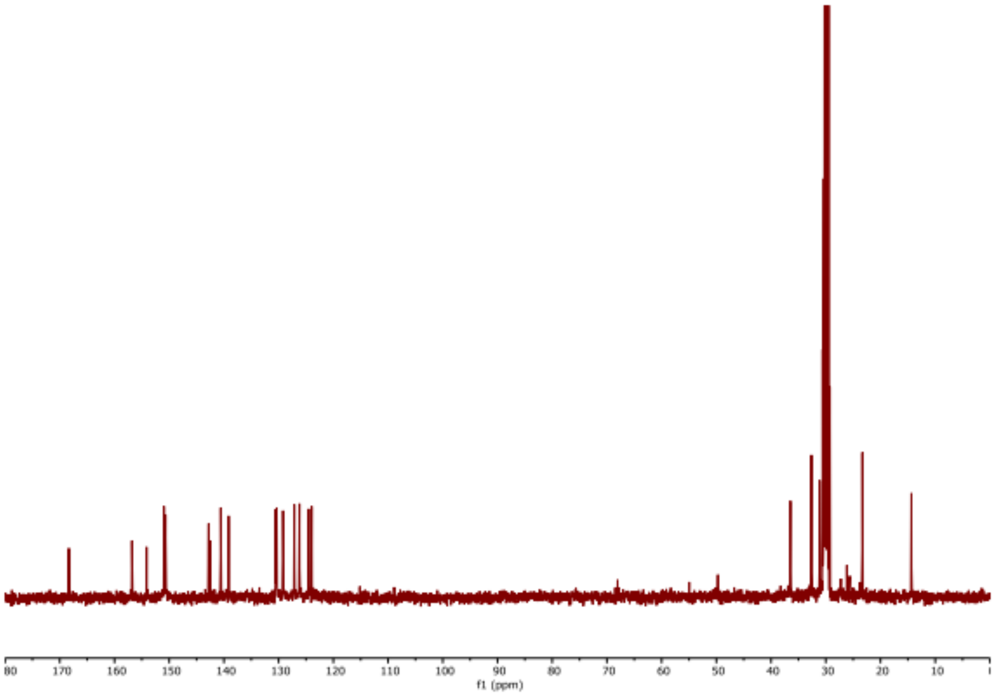

Figure S20: 101 MHz ^{13}C NMR of IrC$_{12}$bpy in acetone-d$_6$.

4. Additional references

1. Ackermann, L.; Novák, P.; Vicente, R.; Hofmann, N., Ruthenium-Catalyzed Regioselective Direct Alkylation of Arenes with Unactivated Alkyl Halides through C-H Bond Cleavage. *Angew. Chem. Int. Ed.* **2009**, *48* (33), 6045-6048.

2. Sprouse, S.; King, K. A.; Spellane, P. J.; Watts, R. J., Photophysical effects of metal-carbon sigma bonds in ortho-metalated complexes of iridium(III) and rhodium(III). *J. Am. Chem. Soc.* **1984**, *106* (22), 6647-6653.

3. Harrad, S.; Wemken, N.; Drage, D. S.; Abdallah, M. A.; Coggins, A.; Perfluoroalkyl Substances in Drinking Water, Indoor Air and Dust from Ireland: Implications for Human Exposure, *Environ. Sci. Technol.* **2019**, *53*, 13449−13457.

4. Haug, L. S.; Salihovic, S.; Jogsten, I. E.; Thomsen, C.; van Bavel, B.; Lindström, G.; Becher, G.; Levels in food and beverages and daily intake of perfluorinated compounds in Norway, *Chemosphere* **2010**, *80*, 1137–1143.

5. Llorca, M.; Farré, M.; Picó, Y.; Müller, J.; Knepper, T. P.; Barceló, D., Analysis of perfluoroalkyl substances in waters from Germany and Spain, *Sci. Total Environ.* **2012**, *431*, 139–150.

6. Cornelis, C.; D'Hollander, W.; Roosens, L.; Covaci, A.; Smolders, R.; Van Den Heuvel, R.; Govarts, E.; Van Campenhout, K.; Reynders, H.; Bervoets, L., First assessment of population exposure to perfluorinated compounds in Flanders, Belgium, *Chemosphere* **2012**, *86* 308–314.

7. Gebbink, W. A.; van Asseldonk, L.; van Leeuwen, S. P. J., Presence of Emerging Per- and Polyfluoroalkyl Substances (PFASs) in River and Drinking Water near a Fluorochemical Production Plant in the Netherlands, *Environ. Sci. Technol.* **2017**, *51*, 11057−11065.

8. Castiglionia, S.; Valsecchib, S.; Polesellob, S.; Rusconib, M.; Melisa, M.; Palmiottoa, M.; Manentic, A.; Davolia, E.; Zuccato, E., Sources and fate of perfluorinated compounds in the aqueous environment and in drinking water of a highly urbanized and industrialized area in Italy, *J. Hazard. Mater.* **2015**, *282*, 51–60.

9. Domingo, J. L., Ericson-Jogsten, I.; Perello, G.; Nadal, M.; Van Bavel, B.; Kaٰrman, A., Human Exposure to Perfluorinated Compounds in Catalonia, Spain: Contribution of Drinking Water and Fish and Shellfish, *J. Agric. Food Chem.* **2012**, *60*, 4408−4415.

10. Boone, J. S.; Vigo, C.; Boone, T.; Byrne, C.; Ferrario, J.; Benson, R.; Donohue, J.; Simmons, J. E.; Kolpin, D. W.; Furlong, E. T.; Glassmeyer, S. T., Per- and polyfluoroalkyl substances in source and treated drinking waters of the United States, *Sci. Total Environ.* **2019**, *653*, 359–369.

11. Li, Y.; Li, J.; Zhang, L.; Huang, Z.; Liu, Y.; Wu, N.; He, J.; Zhang, Z.; Zhang, Y.; Niu, Z., Perfluoroalkyl acids in drinking water of China in 2017: Distribution characteristics, influencing factors and potential risks, *Environ. Int.* **2019**, *123*, 87-95.
